# Identification of compound heterozygous *DNAH11* variants in a Han‐Chinese family with primary ciliary dyskinesia

**DOI:** 10.1111/jcmm.16866

**Published:** 2021-08-18

**Authors:** Ying Xiong, Hong Xia, Lamei Yuan, Sheng Deng, Zerui Ding, Hao Deng

**Affiliations:** ^1^ Center for Experimental Medicine The Third Xiangya Hospital Central South University Changsha China; ^2^ Department of Emergency The Third Xiangya Hospital Central South University Changsha China; ^3^ Disease Genome Research Center Central South University Changsha China; ^4^ Department of Pharmacy Xiangya Hospital Central South University Changsha China; ^5^ Department of Neurology The Third Xiangya Hospital Central South University Changsha China

**Keywords:** compound heterozygous variants, *DNAH11*, minigene assay, primary ciliary dyskinesia, whole‐exome sequencing

## Abstract

Primary ciliary dyskinesia (PCD) is a group of genetically and clinically heterogeneous disorders with motile cilia dysfunction. It is clinically characterized by oto‐sino‐pulmonary diseases and subfertility, and half of the patients have *situs inversus* (Kartagener syndrome). To identify the genetic cause in a Han‐Chinese pedigree, whole‐exome sequencing was conducted in the 37‐year‐old proband, and then, Sanger sequencing was performed on available family members. Minigene splicing assay was applied to verify the impact of the splice‐site variant. Compound heterozygous variants including a splice‐site variant (c.1974‐1G>C, rs1359107415) and a missense variant (c.7787G>A, p.(Arg2596Gln), rs780492669), in the dynein axonemal heavy chain 11 gene (*DNAH11*) were identified and confirmed as the disease‐associated variants of this lineage. The minigene expression *in vitro* revealed that the c.1974‐1G>C variant could cause skipping over exon 12, predicted to result in a truncated protein. This discovery may enlarge the *DNAH11* variant spectrum of PCD, promote accurate genetic counselling and contribute to PCD diagnosis.

## 1 INTRODUCTION

Primary ciliary dyskinesia (PCD, OMIM 244400) is a group of genetically and clinically heterogeneous conditions with ultrastructural and/or functional defects of cilia, which may result in oto‐sino‐pulmonary diseases, subfertility and situs anomalies.^1^, ^2^, ^3^ Estimated PCD prevalence is 1 per 10,000–20,000 with no apparent racial or gender distinctions.^4^, ^5^, ^6^ The true prevalence may be higher than estimated due to diagnostic limitations.^1^, ^5^ Its earliest description in the medical literature is believed to be in 1933 by M. Kartagener, who reported a triad of chronic sinusitis, bronchiectasis and *situs inversus*, following termed Kartagener syndrome (KS).^2^, ^7^ About 50% of all PCD cases display *situs inversus*, which is caused by nodal cilia dysfunction during embryogenesis.^8^, ^9^ Respiratory distress, nasal and pulmonary symptoms usually appear during neonatal period and then gradually progress and eventually develop into bronchiectasis.^10^ Additionally, most PCD patients have non‐chest symptoms, such as recurrent otitis media, chronic rhinitis, recurrent sinusitis, ectopic pregnancy and infertility.^3^, ^11^

PCD is a genetic disease inherited predominantly in an autosomal recessive fashion.^12^ However, X‐linked inheritance has occasionally been evidenced.^13^, ^14^ To date, over 40 PCD‐associated genes have been identified,^15^ accounting for more than 70% of all patients.^12^ Plenty of studies on the genetics and biology of PCD over the last decade allowed the identification of numerous pathogenic variants in genes encoding proteins essential for cilia motility and helped to reveal the underlying pathogenic mechanisms (Table S1).^16^, ^17^, ^18^ Biallelic variants in the dynein axonemal heavy chain 11 gene (*DNAH11*, OMIM 603339) have been reported as responsible for primary ciliary dyskinesia‐7 (OMIM 611884).^15^, ^19^ At least 137 PCD‐associated *DNAH11* variants have been reported, about 70% of which are missense/nonsense variants. The reported *DNAH11* variants and their heterogeneous PCD‐associated phenotypes were listed in Table S2.^19^

In this study, a three‐generation Han‐Chinese family with PCD, consistent with KS’s diagnostic criteria, was recruited. Compound heterozygous variants, c.1974‐1G>C and c.7787G>A (p.(Arg2596Gln)), in *DNAH11* gene (NG_012886.2, NM_001277115.2) were identified as disease‐associated variants.

## 2 MATERIALS AND METHODS

### 2.1 Subjects and clinical data

**FIGURE 1:**
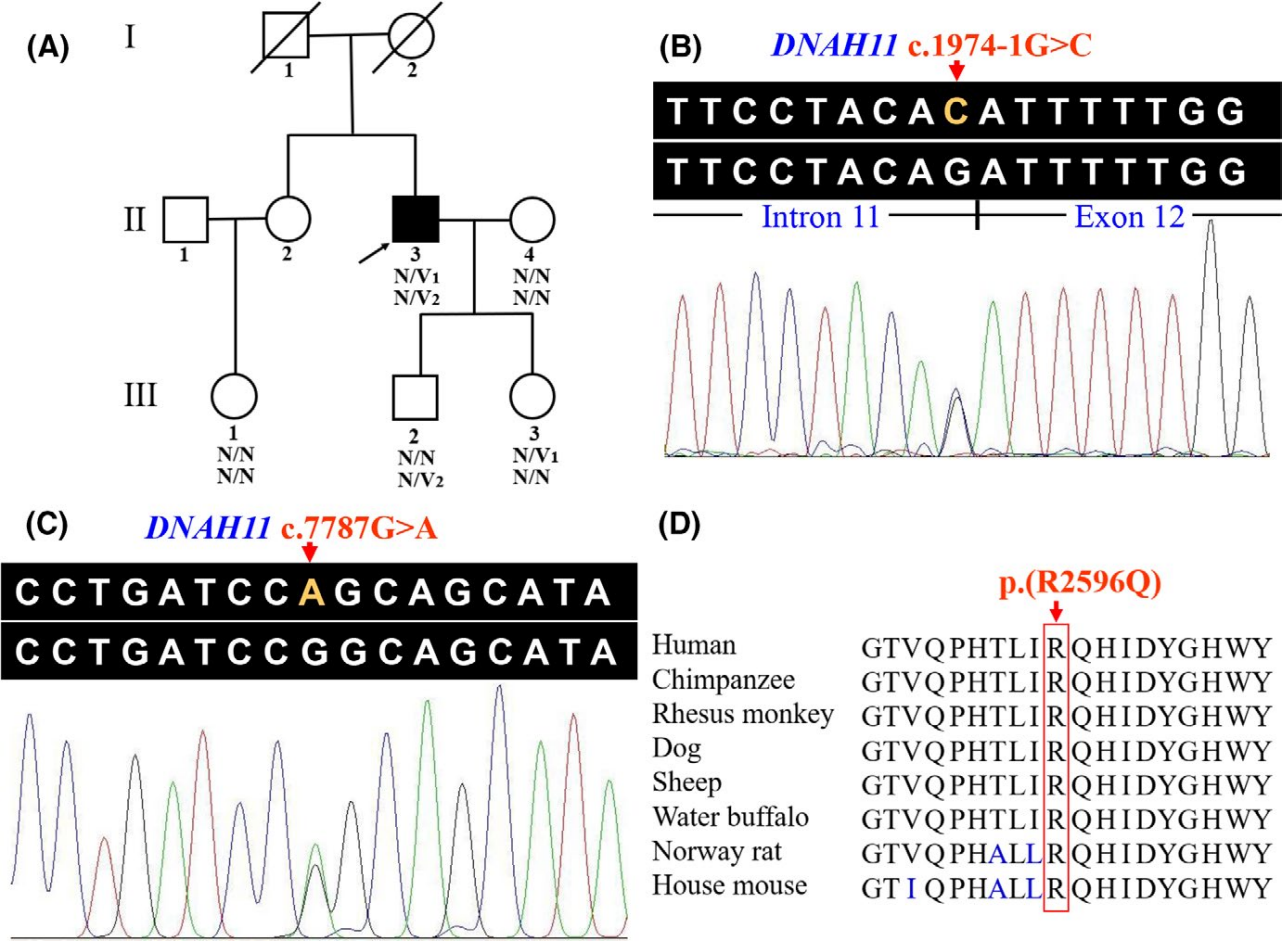
Pedigree and sequence analysis of a Chinese family with PCD. (A) Pedigree with PCD. The proband is indicated by an arrow and solid square. Deceased family members are shown as slashed symbols. N, normal; V_1_, the *DNAH11* c.1974‐1G>C variant; V_2_, the *DNAH11* c.7787G>A variant. (B) The *DNAH11* c.1974‐1G>C variant in the proband. (C) The *DNAH11* c.7787G>A variant in the proband. (D) Conservation analysis of the DNAH11 p.R2596 residue. PCD, primary ciliary dyskinesia; *DNAH11*, the dynein axonemal heavy chain 11 gene; R, arginine; Q, glutamine

A three‐generation Chinese family with PCD was recruited from south central China (Figure 1A), and an unrelated healthy male without related condition and family history was enrolled as a control. After informed the relevant matters and obtained signed consent from each enrolee, clinical data and peripheral blood specimens were acquired from five available family members, comprised of the proband (II:3) and four unaffected individuals (II:4, III:1, III:2 and III:3), as well as the control. The PCD diagnosis was based on clinical features, the score of Primary Ciliary Dyskinesia Rule (PICADAR), auxiliary examinations including nasal nitric oxide (nNO) measurement, nasal endoscopy, acoustic immittance testing, pure tone audiometry, paranasal sinus and chest computed tomography (CT), and pulmonary function tests, as well as genetic testing.^3^, ^20^ The family rejected transmission electron microscopy (TEM), high‐speed video microscopy (HSVM) and immunofluorescence due to their psychological fear. This research plan was approved by the Institutional Review Board of the Third Xiangya Hospital, Central South University, Changsha, China. All studies were performed in accordance with the Declaration of Helsinki.

### 2.2 Whole‐exome sequencing

Genomic DNA (gDNA) was extracted from peripheral blood samples via standard procedure previously reported.^21^ One microgram of gDNA from the proband (II:3) was randomly broken into fragments by Covaris technology, and 150–250 bp of fragments were selected.^22^ Size‐selected fragments were subjected to end‐repairing, A‐tailing and ligating adaptors. Ligation‐mediated polymerase chain reaction (PCR) amplified the ligated fragments. Purification and hybridization to the exome array for enrichment were performed.^23^ DNA nanoballs were formed via rolling circle amplification using circular single‐stranded libraries and then loaded onto a sequencing chip. The captured exome library underwent high‐throughput sequencing on the BGISEQ‐500 (BGI, Shenzhen, China) platform in accord with the manufacturer's instructions.^24^ Sequencing‐derived raw image files obtained were transformed into ‘raw data’ with BGISEQ‐500 base calling software.

### 2.3 Variant analysis

To obtain high‐quality sequencing data, the clean data were filtered from ‘raw data’ following the strict data filtering criteria and data analysis quality control setup. The following criteria to remove reads were used in ‘data cleanup’ process: (1) adaptor sequence, (2) low‐quality base (base quality ≤5) ratios more than 50% and (3) unknown base ratios more than 10%. All clean reads were aligned to the human reference genome (GRCh37/hg19, http://genome.ucsc.edu/) by Burrows‐Wheeler Aligner (BWA, v0.7.15).^25^ Duplicate sequence reads were marked by Picard tools (v2.5.0; http://broadinstitute.github.io/picard/). Local realignment around insertions‐deletions (Indels) and base quality score recalibrations were conducted with the Genome Analysis Toolkit (GATK, v3.7; https://www.broadinstitute.org/gatk/guide/best‐practices).^23^ Data analysis quality control process was rigidly conducted during the bioinformatics analysis pipeline. HaplotypeCaller of GATK (v3.7) detected genomic variation, including single nucleotide polymorphisms (SNPs) and Indels. The SnpEff software (http://snpeff.sourceforge.net/SnpEff_manual.html) was then used to annotate variants. The filtering flowsheet is presented in Figure S1. All candidate variants were filtered by several public databases, including the NHLBI exome sequencing project (ESP) 6500, 1000 Genomes Project, the Single Nucleotide Polymorphism database (dbSNP, version 141), Genome Aggregation Database (gnomAD) and the Exome Aggregation Consortium (ExAC), and the in‐house exome database. The variants with the minor allele frequency ≥0.01 were removed. After excluding synonymous variants, non‐exonic SNPs and non‐canonical splicing sites, non‐synonymous variants in the 42 PCD‐associated genes were selected.^26^ The predicted pathogenicity of selected homozygous or compound heterozygous variants was conducted on MutationTaster (http://www.mutationtaster.org/), Sorting Intolerant from Tolerant (SIFT, http://sift.jcvi.org/), Polymorphism Phenotyping version 2 (PolyPhen‐2, http://genetics.bwh.harvard.edu/pph2/) and Berkeley Drosophila Genome Project/Splice Site Prediction by Neural Network (BDGP/NNSplice, http://www.fruitfly.org/seq_tools/splice.html).^24^ Sanger sequencing of enrolled family members verified the identified potential causative variants with an ABI3500 sequencer (Applied Biosystems Inc.). The following are the primer sequences for potential variants in *DNAH11* gene designed by Primer3 (http://primer3.ut.ee/): 5′‐GCAAAAGCAATTAATACGCACA‐3′ and 5′‐TCAAATTGATCAAGCAAAGTGG‐3′, as well as 5′‐AAAAATTGATTTATTTTATCGACGAC‐3′ and 5′‐TGGGGATAATTGCACTTGAA‐3′.

Conservation analyses among multiple diverse species were employed by the NCBI Basic Local Alignment Search Tool (BLAST, https://blast.ncbi.nlm.nih.gov/Blast.cgi).^22^

Possible pathogenic effects of candidate variants were further predicted by the Protein Variation Effect Analyzer (PROVEAN, http://provean.jcvi.org/index.php), NetGene2 (http://www.cbs.dtu.dk/services/NetGene2/) and Spliceman (http://fairbrother.biomed.brown.edu/spliceman/).^27^ Wild‐type and mutant protein of tertiary structure prediction were conducted with online SWISS‐MODEL tool (http://www.swissmodel.expasy.org) and further visualized via PyMOL software (version 2.3; Schrödinger, LLC, Portland, USA).^28^ The classification of the identified variants was carried out by the American College of Medical Genetics (ACMG) interpretation guidelines for variants in Mendelian disorders.^29^

### 2.4 Minigene splicing assay

The minigene regions spanning *DNAH11* exon 11–13 and intron 11–12 of the *DNAH11* gene were amplified from gDNA of the control using a forward primer *DNAH11*‐F (5′‐AAGCTTGGTACCGAGCTCGGATCCATTGAATGTGGTCATGTAGTTCTTAACA‐3′) with the restriction site *Bam*HI and a reverse primer *DNAH11*‐R (5′‐TTAAACGGGCCCTCTAGACTCGAGCTTTAAAATAGTGTTCCTTTTCTTGAAG‐3′) with the restriction site *Xho*I. By using ClonExpress II One Step Cloning Kit (Vazyme, Nanjing, China), the amplified products were cloned into the pMini‐CopGFP vector (Beijing Hitrobio Biotechnology Co., Ltd.). The wild‐type plasmid was validated by Sanger sequencing. The mutant fragments were obtained with mutagenesis primers of *DNAH11*‐MT‐F (5′‐CCTACACATTTTTGGGCAATCCTGATCACGCT‐3′) and *DNAH11*‐MT‐R (5′‐GCCCAAAAATGTGTAGGAATAAGGTTTTTCTACTGGTTT‐3′). The mutant plasmid was validated by Sanger sequencing. The selected plasmids were prepared for further transfection. Human embryonic kidney 293T (HEK293T) cells were cultured in Dulbecco's modified Eagle's medium supplement with 10% foetal bovine serum (HyClone) and incubated at 37℃ and 5% CO_2_. The recombinant plasmids were transiently transfected into HEK293T cells with Lipofectamine 2000 (Invitrogen) following the instructions. Total RNA was extracted from cells cultured for 48 h with TRIzol reagent (Cowin Biotech Co.). Reverse transcription‐polymerase chain reaction (RT‐PCR) was conducted with a pair primer of MiniRT‐F (5′‐GGCTAACTAGAGAACCCACTGCTTA‐3′) and MiniRT‐R (5′‐GTTTAAACGGGCCCTCTAGACTCGA‐3′). PCR fragments were analysed by agarose gel electrophoresis, and isoforms were determined by Sanger sequencing.

## 3 RESULTS

### 3.1 Clinical findings

**FIGURE 2:**
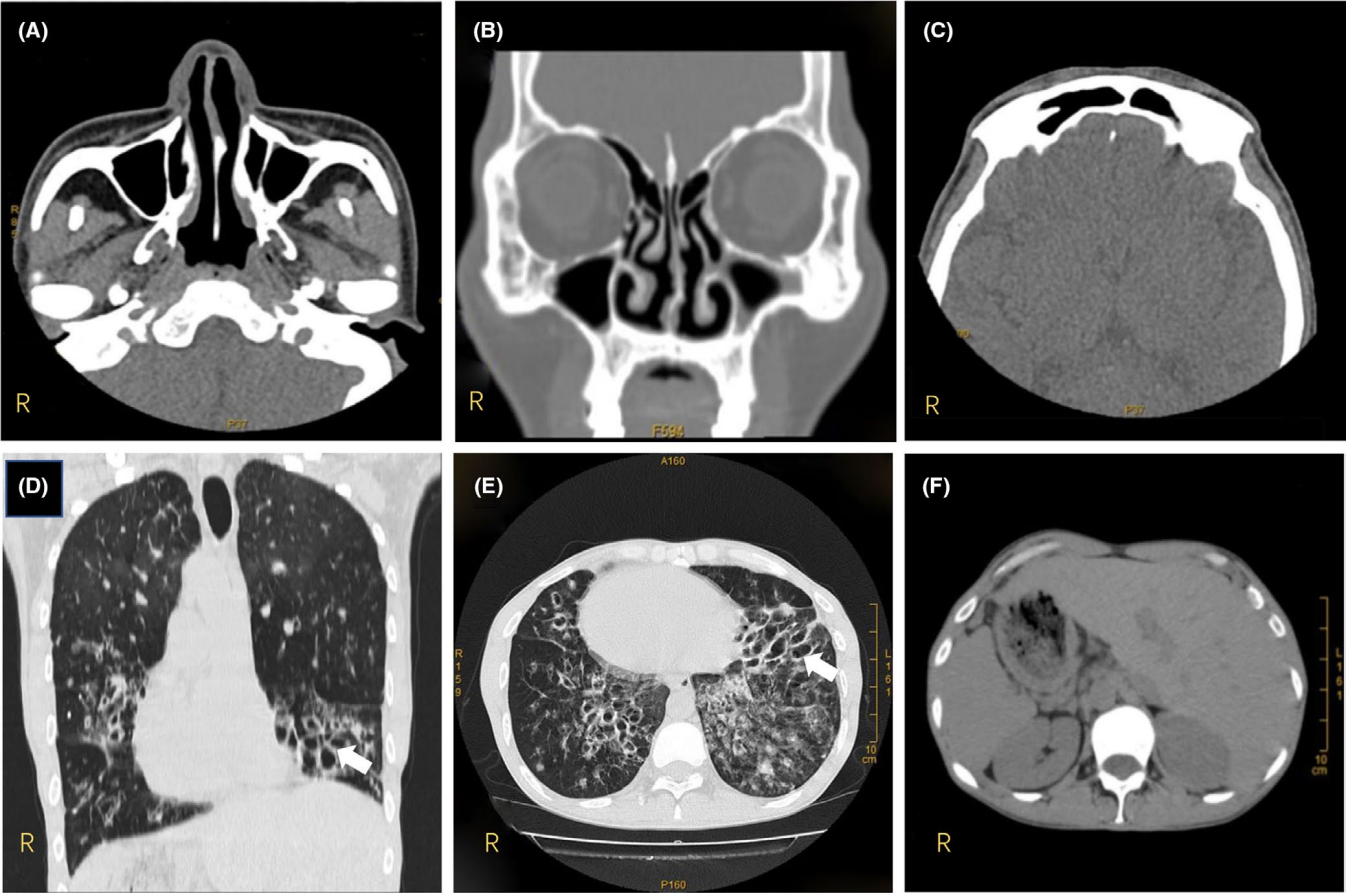
CT scan of the PCD proband (II:3). The paranasal sinus CT scan of the proband shows non‐specific thickening of the mucosa on the bilateral maxillary sinuses (A, B) and left frontal sinus (C). The chest CT scan shows multiple patchy high‐density shadows in bilateral lungs with blurred boundaries, bronchial dilatation, thickened bilateral bronchial walls (D, E) and *situs inversus* (D, E, F). Bronchiectasis is indicated by white arrows (D, E). CT, computed tomography; PCD, primary ciliary dyskinesia

The proband, a 37‐year‐old male, had been clinically diagnosed with PCD based on the clinical features by two experienced respiratory physicians from the Third Xiangya Hospital, Central South University. At recruitment, he presented with the chief complaints of wet cough for 20 years and had intermittent fever in the last week. Physical examinations revealed a right‐side apical pulse, scattered rhonchi and coarse crackles on both lung fields. He was born with *situs inversus*. During childhood, he developed chronic rhinitis, recurrent sinusitis, frequent pneumonias and ear infections, as well as hearing impairment. At the age of 29 years, he was diagnosed with chronic obstructive pulmonary disease from his recurrent respiratory tract infections. Subfertility was denied and he had two children (III:2 and III:3). His PICADAR score was 6 points (cut‐off score of 5 points with a sensitivity of 90% and a specificity of 75%).^30^ The result of nNO level was 141 ppb, which is below the 200 ppb cut‐off score.^31^ Pulmonary function tests revealed a forced expiratory volume in 1 s (FEV_1_) of 1.06 L, a forced vital capacity (FVC) of 2.15 L and an FEV_1_/FVC ratio of 49.36%. He was deemed to have otitis media with effusion and bilateral moderate hearing loss by otolaryngological examination. Paranasal sinus three‐dimensional imaging showed non‐specific mucosal thickening on the bilateral maxillary sinuses (Figure 2A, B) and the left frontal sinus (Figure 2C). Chest CT presented multiple patchy high‐density shadows in bilateral lungs with blurred boundaries, bronchial dilatation, thickened bilateral bronchial walls (Figure 2D, E) and *situs inversus* (Figure 2D, E, F). Detailed clinical characteristics of available members in this family are presented in Table 1.

**TABLE 1:** Clinical features and genotypes of available members in this PCD family

Subject	II:3	II:4	III:1	III:2	III:3
Sex	M	F	F	M	F
Age (years)	37	30	25	9	6
Age at onset (years)	29	−	−	−	−
Zygosity	Compound heterozygote	Wild type	Wild type	Heterozygote	Heterozygote
*DNAH11* variant	c.1974‐1G > C c.7787G > A	−	−	c.7787G > A	c.1974‐1G > C
Chronic rhinitis	+	−	−	−	−
Recurrent sinusitis	+	−	−	−	−
Chronic otitis media	+	−	−	−	−
Hearing impairment	+	−	−	−	−
Bronchiectasis	+	−	−	−	−
*Situs inversus*	+	−	−	−	−
Infertility	−	−	Unknown	Unknown	Unknown

Abbreviations: −, absent; +, present; *DNAH11*, the dynein axonemal heavy chain 11 gene; F, female; M, male; PCD, primary ciliary dyskinesia.

### 3.2 Molecular genetic analysis

Whole‐exome sequencing results reported that the gDNA of proband generated 15,255.13 Mb raw data. After data filtering, there were 133.06 million total effective reads with 99.92% mapped to the human reference sequence. Average target region sequencing depth was 171.08× which guaranteed enough accuracy to call variants in 99.42% of the target regions covered >10×. A total of 92,516 SNPs and 14,363 Indels were detected. A preferential scheme following previous studies identified the pathogenic variants in the proband.^32^, ^33^ The variants in the NHLBI ESP6500 and 1000 Genomes Project, with a minor allele frequency of ≥1%, were removed. The remaining sequence variants were filtered by the BGI in‐house exome database (2375 controls). Considering the autosomal recessive model, only compound heterozygous variants (c.1974‐1G>C and c.7787G>A) in the *DNAH11* gene, a known PCD gene, were suspected as the disease‐associated variants for the proband, and no other variants in known PCD‐associated genes were detected. The *DNAH11* c.1974‐1G>C splice‐site variant was absent from NHLBI ESP6500, 1000 Genomes Project, the BGI in‐house exome database and our in‐house exome database (697 controls). It has been recorded in the dbSNP (rs1359107415) and has a very low frequency in the global population of gnomAD (0.000007). The missense variant c.7787G>A (p.(Arg2596Gln)) has been recorded in the dbSNP (rs780492669). It has a very low frequency in the global population of ExAC (0.00002) and gnomAD (0.000014). The results of Sanger sequencing (Figure 1B, C) showed that the compound heterozygous variants co‐segregated with the clinical phenotype in this family. This suggests that the compound heterozygous variants (c.1974‐1G>C and c.7787G>A) were responsible for this family's PCD.

### 3.3 Bioinformation analysis

**FIGURE 3:**
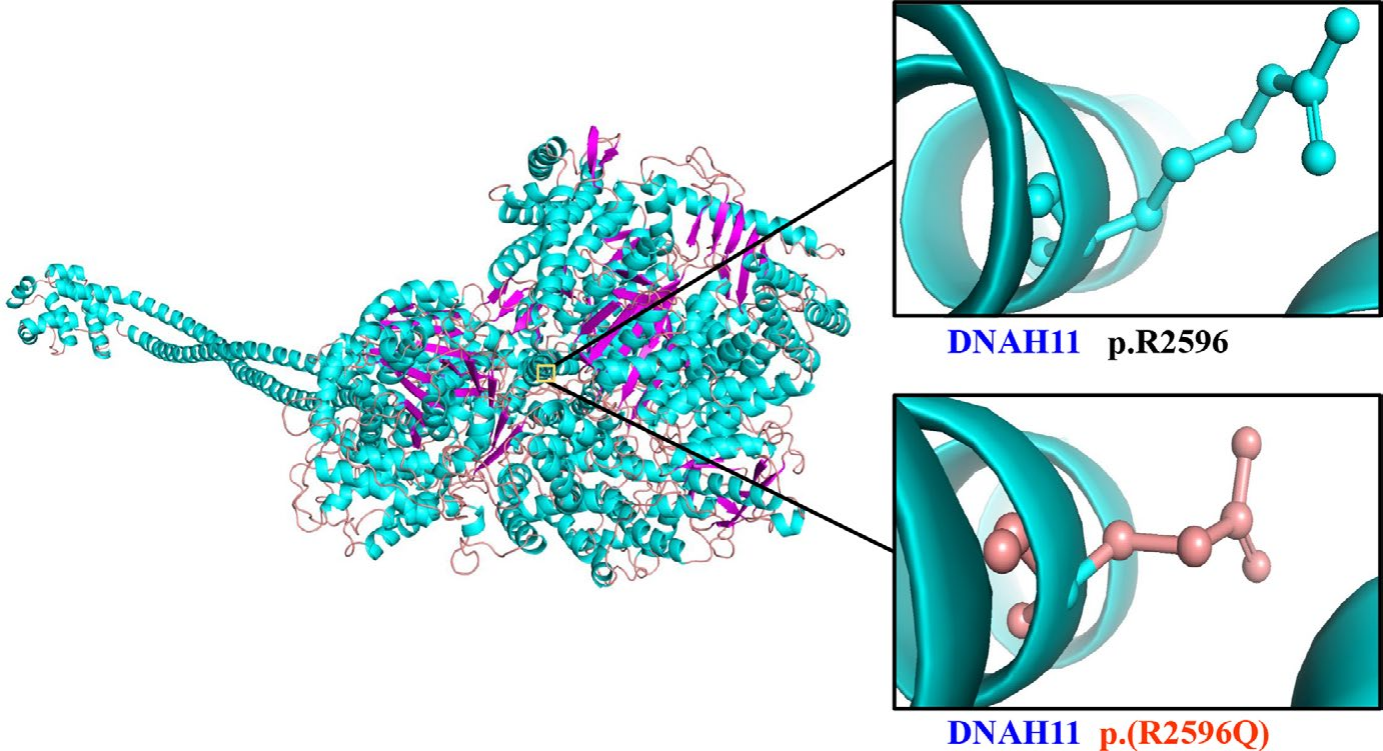
Cartoon model of the DNAH11 protein structure (residues) by PyMOL based on the SWISS‐MODEL. The arginine and mutated glutamine at position 2596 are indicated with ball‐and‐stick models. R, arginine; Q, glutamine

Conservative analysis results revealed that the arginine at site 2596 (p.R2596) was highly conserved in many species (Figure 1D). Structural modelling showed the conformational changes at residue 2596 in which arginine, a positively charged, basic amino acid, was substituted by glutamine, a neutral amino acid (Figure 3), which may influence the solubility and specificity in molecular interaction of the whole protein.^34^ The c.7787G>A (p.(Arg2596Gln)) variant in the *DNAH11* gene was predicted to be deleterious by MutationTaster, PROVEAN, SIFT (0.000, damaging) and PolyPhen‐2 (0.999, probably damaging). BDGP/NNSplice and NetGene2 predicted that the splice‐site variant in *DNAH11* (c.1974‐1G>C, intron 11) would destroy the acceptor site. The splice‐site variant was also predicted to disrupt splicing process by Spliceman, in which the calculated L1 distance was 35,575 with a prediction score of 70%. The predicted results of functional prediction software programs are shown in Table 2. Based on the ACMG guidelines, the *DNAH11* c.1974‐1G>C and c.7787G>A variants were categorized as ‘pathogenic’ and ‘likely pathogenic’, respectively.

**TABLE 2:** The predicted results of c.1974‐1G > C and c.7787G > A variants in the *DNAH11* gene

Variant	Type	MutationTaster	PROVEAN	SIFT	PolyPhen‐2	BDGP/NNSplice	NetGene2	Spliceman
c.1974‐1G > C	Splice‐site	−	−	−	−	Loss of acceptor splice‐site^a^	Loss of acceptor splice‐site^a^	L1: 35 575; ranking: 70%^b^
c.7787G > A	Missense	~1 (Disease causing)	−3.770 (Deleterious)	0.000 (Damaging)	0.999 (Probably damaging)	−	−	−

Abbreviations: −, not applicable; BDGP/NNSplice, Berkeley Drosophila Genome Project/Splice Site Prediction by Neural Network; *DNAH11*, the dynein axonemal heavy chain 11 gene; L1, the L1 distance calculated with Spliceman; PolyPhen‐2, Polymorphism Phenotyping version 2; PROVEAN, Protein Variation Effect Analyzer; SIFT, Sorting Intolerant from Tolerant.

^a^ Destroy the acceptor splice‐site.

^b^ Likely disrupt splicing.

### 3.4 Splicing study of DNAH11 c.1974‐1G>C by minigene assay

**FIGURE 4:**
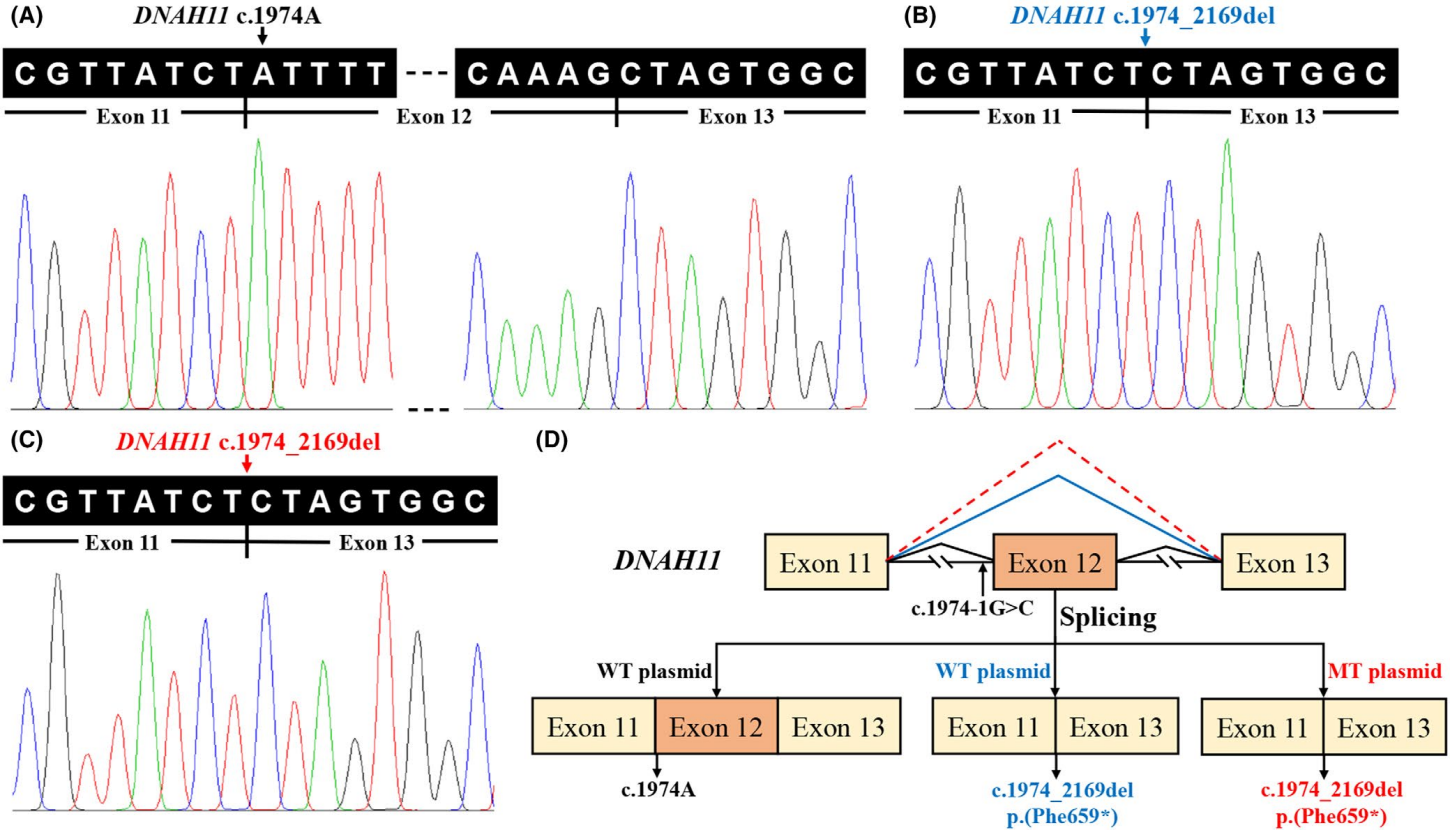
Minigene assay for *DNAH11* c.1974‐1G>C variant and schematic diagram of splicing pattern. (A) Sanger sequencing of the normal splicing isoform for WT plasmid consistent with *DNAH11* exon 11–13. (B) Sanger sequencing of the aberrant splicing for WT plasmid consistent with *DNAH11* exon 11 and exon 13. (C) Sanger sequencing of the aberrant splicing for MT plasmid consistent with *DNAH11* exon 11 and exon 13. (D) Schematic diagram of the *DNAH11* c.1974‐1G>C variant. *DNAH11*, the dynein axonemal heavy chain 11 gene; WT, wild‐type; MT, mutant

Minigene analysis was performed to further characterize the abnormal splicing. Agarose gel electrophoresis of RT‐PCR products showed two bands from the wild type and a single band in the mutant type. Sanger sequencing revealed a normal splicing isoform for wild type, consistent with *DNAH11* exon 11–13 (Figure 4A), and an aberrant splicing for wild type and mutant type, consistent with *DNAH11* exon 11 and 13 (Figure 4B, C). The minigene analysis suggested that the c.1974‐1G>C substitution can abrogate the intron 11 canonical acceptor splice site and lead to the skipping of exon 12 (c.1974_2169del), predicted to result in a truncated protein (p.(Phe659*)) (Figure 4D).

## 4 DISCUSSION

PCD is a rare genetic disease predominantly inherited in an autosomal recessive mode and resulted from impaired ciliary function.^4^, ^35^ At present, specific clinical treatment for PCD is not unravelled, and the clinical curative effect extremely depends on early clinical diagnosis and timely intervention.^5^, ^36^ Gene editing in respiratory ciliated cells with the *DNAH11* variant, c.6727C>T, p.(R2243*), can restore cilia motility *ex vivo*, indicating an exploitable strategy for future therapy.^37^ The classical PCD diagnosis was cilia ultrastructural defects visualized by TEM.^38^ However, normal cilia ultrastructure appeared in about 30% patients,^39^ ~20% of which had biallelic *DNAH11* variants.^40^ The tests of TEM, HSVM and immunofluorescence applied in PCD patients show significant efficacy in pathological changes, as well as prospect in further genotype‐phenotype relationship with genetic discovery.^41^, ^42^ With the emerging molecular genetic analysis, the diagnostic yield can be obviously raised.^38^, ^42^

In this study, compound heterozygous variants (c.1974‐1G>C and c.7787G>A) in the *DNAH11* gene were identified in the PCD patient via whole‐exome sequencing and Sanger sequencing, and the heterozygous *DNAH11* c.1974‐1G>C and c.7787G>A (p.(Arg2596Gln)) variants were detected in his unaffected daughter and son, respectively. These variants co‐segregated with the family's PCD phenotype. The two variants with a low frequency in the general population were unidentified in the 2375 Chinese controls from the BGI exome database and 697 ethnically matched controls from our in‐house exome database. *In silico* analysis predicted the splice‐site c.1974‐1G>C variant to impair splicing, involving in the conserved AG dinucleotide. The interested splicing effect of the c.1974‐1G>C variant was further explored by complementary DNA analysis with total RNA extracted from the control's and proband's (II:3) lymphocytes. However, no *DNAH11* gene expression was detected in the RNA samples from the control or proband (data not shown), consistent with the relatively low *DNAH11* expression in the lymphocytes (BioGPS, http://biogps.org).^43^ Due to the unavailability of specific tissues for related respiratory epithelial cells, we next tried to evaluate the splicing impact by minigene assay *in vitro*. The wild‐type plasmid seems to generate two transcripts, including the wild type by normal splicing and the shorter one observed in the mutant plasmid by aberrant splicing, which may correspond to alternative splicing using alternative sites not characterized in humans. However, the canonical transcript was not detected in the mutant plasmid. The findings of minigene assay should also be cautiously explained as it is different from the actual process *in vivo*, especially in affected tissues. The *in vitro* splicing analysis cannot fully mimic the *in vivo* conditions, such as cell‐ or tissue‐dependent splicing factors, transcription rate, chromatin state, isoform balance and intron size.^44^, ^45^ At least, our studies showed the different splicing patterns in wild‐type and mutant plasmids, indicating that the c.1974‐1G>C variant contributed to the discard of the 3’ accepter splice site in intron 11, leading to exon 12 skipping, and a predicted truncated protein. Taken together with *in silico* predicted effect of the c.7787G>A (p.(Arg2596Gln)) variant and high evolutionary conservation of p.R2596, the above factors indicate the possible pathogenic effect of these compound heterozygous *DNAH11* variants in this family. Further functional studies and co‐segregation analyses in more PCD families are warranted to validate the accurate pathogenicity of those variants and genotype‐phenotype correlation.

The unimplemented cilia ultrastructural or function assessment may be the limitations of our study. Previous studies showed that in most PCD cases (92%) with *DNAH11* variants, no specific ciliary ultrastructural abnormalities were detected by conventional TEM (Table S2). However, subtle outer dynein arm defects in the proximal ciliary part were visualized when re‐examined by three‐dimensional electron tomography and immunofluorescence microscopy in two studies.^42^, ^46^ No significant genotype‐phenotype correlation seems to exist in PCD individuals with *DNAH11* variants (Table S2). Intra‐familial patients carrying the same *DNAH11* variant may present considerable phenotypic variability, which may be influenced by both intrinsic and extrinsic factors, such as modifier genes, environmental factors and timely valid therapeutics.^19^, ^42^, ^47^

The *DNAH11* gene, mapped to chromosome 7p15.3, comprises 82 exons spanning over 358 kb and yielding a 14 kb mRNA.^37^, ^48^ DNAH11 protein, a member of the dynein heavy chain family, is involved in the composition of the axonemal dynein complex, which transforms energy into mechanical force, submits it to the microtubule and generates power to move the cilia and sperm flagella.^19^, ^49^ Some KS or PCD male patients may suffer from asthenozoospermia.^50^ In our study, compound heterozygous variants, c.1974‐1G>C and c.7787G>A, in *DNAH11* gene were identified in the proband who has two children, heterozygous variant carriers. The escape of infertility may be due to the compensation of other homologous protein(s) or little effect on sperm production of the variants.

DNAH11 is composed of 4516 amino acids with the important globular head or motor domain. An N terminus, six ATPases associated with diverse cellular activity domains (AAA domains), a microtubule‐binding region, two coiled‐coil segments and a C terminus constitute the protein.^40^, ^48^ Each of the first four AAA domains contains a triphosphate‐binding loop, also known as the P‐loop (P1‐P4).^51^ The P1 site is the primary hydrolysis site, while the adjacent P‐loop domains influence the P1 site activity and the microtubule‐binding domain attachment.^52^

The absence of the *Chlamydomonas reinhardtii* β‐dynein heavy chain, which is a ortholog of DNAH11 in human respiratory cilia, reduces both gliding velocity and ATPase activity, supporting an essential role for motility.^41^, ^53^ Though the penetrance was modified by genetic background, the mouse harbouring the *Dnah11* gene c.6811G>A (p.Glu2271Lys) variant presents left‐right body patterning asymmetry, static respiratory cilia with normal ultrastructural appearance and reduced sperm motility, accompanied by rhinitis, sinusitis and otitis media, a viable model of human PCD.^54^, ^55^

The identified c.7787G>A missense variant, located in exon 47, leads to the highly conserved arginine at position 2596 being substituted by glutamine (p.(Arg2596Gln)). This variant is positioned on the P3 of the third AAA domain, related to dynein function and microtubule binding.^52^ Similarly, an adjacent missense variant, c.7772C>T, p.(Pro2591Leu), in the compound heterozygous state (with c.8698C>T, p.(Arg2900*)), positioned at the same segment, changing proline to leucine in the third AAA domain, has been reported as a disease‐causing allele for PCD in a white Caucasian patient.^54^ These two PCD patients shared some clinical characteristics, including persistent wet cough, rhinitis, otitis media and situs anomalies. Six AAA domains, composing the motor domain, are linked together as a hexamer in a ring shape, which couples ATP binding and hydrolysis to conformational change.^56^, ^57^ Although currently the definite origin of AAA domains remains veiled, it can be inferred that the various individual AAA domains would give rise to changes in composition and structure of hexamer, eventually dynein motility.^56^

In conclusion, compound heterozygous *DNAH11* variants, including a splice‐site variant (c.1974‐1G>C) and a missense variant (c.7787G>A, p.(Arg2596Gln)), may be the genetic basis for the PCD in this Han‐Chinese family. The present study first reports those compound heterozygous variants acting as a potential disease‐causing factor in PCD. The findings may broaden the spectrum of PCD variants and facilitate pinpoint diagnosis and prognosis. Further functional studies may help to reveal the underlying mechanisms of *DNAH11* gene variant inducing PCD and provide possible specific therapies in the future.

## CONFLICT OF INTEREST

The authors confirm that there are no conflicts of interest.

## AUTHOR CONTRIBUTIONS

**Hao Deng:** Conceptualization (equal); Data curation (equal); Funding acquisition (equal); Project administration (equal); Supervision (equal); Validation (equal); Writing‐review & editing (equal). **Ying Xiong:** Conceptualization (equal); Data curation (equal); Methodology (equal); Software (equal); Visualization (equal); Writing‐original draft (equal); Writing‐review & editing (equal). **Hong Xia:** Conceptualization (equal); Funding acquisition (equal); Methodology (equal); Visualization (equal); Writing‐original draft (equal); Writing‐review & editing (equal). **Lamei Yuan:** Data curation (equal); Funding acquisition (equal); Methodology (equal); Supervision (equal); Validation (equal); Writing‐review & editing (equal). **Sheng Deng:** Data curation (equal); Formal analysis (equal); Funding acquisition (equal); Methodology (equal). **Zerui Ding:** Data curation (equal); Software (equal); Visualization (equal).

## Supporting information

Fig S1

Table S1

Table S2

## Data Availability

All data generated or used during the study are available from the corresponding author upon reasonable request.

## References

[1] Knowles MR, Daniels LA, Davis SD, et al. Primary ciliary dyskinesia. Recent advances in diagnostics, genetics, and characterization of clinical disease. Am J Respir Crit Care Med. 2013;188(8):913‐922. 23796196 10.1164/rccm.201301-0059CI PMC3826280

[2] Takeuchi K, Kitano M, Ishinaga H, et al. Recent advances in primary ciliary dyskinesia. Auris Nasus Larynx. 2016;43(3):229‐236. 26527516 10.1016/j.anl.2015.09.012

[3] Lucas JS, Barbato A, Collins SA, et al. European Respiratory Society guidelines for the diagnosis of primary ciliary dyskinesia. Eur Respir J. 2017;49(1):1601090. 27836958 10.1183/13993003.01090-2016 PMC6054534

[4] Horani A, Ferkol TW. Advances in the genetics of primary ciliary dyskinesia: clinical implications. Chest. 2018;154(3):645‐652. 29800551 10.1016/j.chest.2018.05.007 PMC6130327

[5] Mirra V, Werner C, Santamaria F. Primary ciliary dyskinesia: an update on clinical aspects, genetics, diagnosis, and future treatment strategies. Front Pediatr. 2017;5:135. 28649564 10.3389/fped.2017.00135 PMC5465251

[6] Kuehni CE, Frischer T, Strippoli MP, et al. Factors influencing age at diagnosis of primary ciliary dyskinesia in European children. Eur Respir J. 2010;36(6):1248‐1258. 20530032 10.1183/09031936.00001010

[7] Kartagener M. Zur pathogenese der bronchiektasien. Bronchiektasien bei situs viscerum inversus. Beitr Klin Tuberk Spezif Tuberkuloseforsch. 1933;83:489‐501.

[8] Deng H, Xia H, Deng S. Genetic basis of human left‐right asymmetry disorders. Expert Rev Mol Med. 2015;16:e19. 26258520 10.1017/erm.2014.22

[9] Hirokawa N, Tanaka Y, Okada Y. Left‐right determination: involvement of molecular motor KIF3, cilia, and nodal flow. Cold Spring Harb Perspect Biol. 2009;1(1):a000802. 20066075 10.1101/cshperspect.a000802 PMC2742083

[10] Lucas JS, Burgess A, Mitchison HM, et al. Diagnosis and management of primary ciliary dyskinesia. Arch Dis Child. 2014;99(9):850‐856. 24771309 10.1136/archdischild-2013-304831 PMC4145427

[11] Goutaki M, Meier AB, Halbeisen FS, et al. Clinical manifestations in primary ciliary dyskinesia: systematic review and meta‐analysis. Eur Respir J. 2016;48(4):1081‐1095. 27492829 10.1183/13993003.00736-2016

[12] Horani A, Ferkol TW. Primary ciliary dyskinesia and associated sensory ciliopathies. Expert Rev Respir Med. 2016;10(5):569‐576. 26967669 10.1586/17476348.2016.1165612 PMC4893162

[13] Moore A, Escudier E, Roger G, et al. RPGR is mutated in patients with a complex X linked phenotype combining primary ciliary dyskinesia and retinitis pigmentosa. J Med Genet. 2006;43(4):326‐333. 16055928 10.1136/jmg.2005.034868 PMC2563225

[14] Paff T, Loges NT, Aprea I, et al. Mutations in PIH1D3 cause X‐linked primary ciliary dyskinesia with outer and inner dynein arm defects. Am J Hum Genet. 2017;100(1):160‐168. 28041644 10.1016/j.ajhg.2016.11.019 PMC5223094

[15] Lucas JS, Davis SD, Omran H, et al. Primary ciliary dyskinesia in the genomics age. Lancet Respir Med. 2020;8(2):202‐216. 31624012 10.1016/S2213-2600(19)30374-1

[16] Poprzeczko M, Bicka M, Farahat H, et al. Rare human diseases: model organisms in deciphering the molecular basis of primary ciliary dyskinesia. Cells. 2019;8(12):1614. 10.3390/cells8121614 PMC6952885 31835861

[17] Pennarun G, Escudier E, Chapelin C, et al. Loss‐of‐function mutations in a human gene related to chlamydomonas reinhardtii dynein IC78 result in primary ciliary dyskinesia. Am J Hum Genet. 1999;65(6):1508‐1519. 10577904 10.1086/302683 PMC1288361

[18] Becker‐Heck A, Zohn IE, Okabe N, et al. The coiled‐coil domain containing protein CCDC40 is essential for motile cilia function and left‐right axis formation. Nat Genet. 2011;43(1):79‐84. 21131974 10.1038/ng.727 PMC3132183

[19] Schwabe GC, Hoffmann K, Loges NT, et al. Primary ciliary dyskinesia associated with normal axoneme ultrastructure is caused by DNAH11 mutations. Hum Mutat. 2008;29(2):289‐298. 18022865 10.1002/humu.20656

[20] Shapiro AJ, Davis SD, Polineni D, et al. Diagnosis of primary ciliary dyskinesia. An official American Thoracic Society clinical practice guideline. Am J Respir Crit Care Med. 2018;197(12):e24‐e39. 29905515 10.1164/rccm.201805-0819ST PMC6006411

[21] Chen X, Deng S, Xia H, et al. Identification of a CCDC114 variant in a Han‐Chinese patient with situs inversus. Exp Ther Med. 2020;20(4):3336‐3342. 32855706 10.3892/etm.2020.9059 PMC7444340

[22] Chen X, Deng S, Xu H, et al. Novel and recurring NOTCH3 mutations in two Chinese patients with CADASIL. Neurodegener Dis. 2019;19(1):35‐42. 31212292 10.1159/000500166

[23] Xiang Q, Yuan L, Cao Y, et al. Identification of a heterozygous mutation in the TGFBI gene in a Hui‐Chinese family with corneal dystrophy. J Ophthalmol. 2019;2019:2824179. 30915236 10.1155/2019/2824179 PMC6399521

[24] Deng S, Wu S, Xia H, et al. Identification of a frame shift mutation in the CCDC151 gene in a Han‐Chinese family with Kartagener syndrome. Biosci Rep. 2020;40(6):BSR20192510. 32490514 10.1042/BSR20192510 PMC7298131

[25] Fan K, Zhu H, Xu H, et al. The identification of a transthyretin variant p.D38G in a Chinese family with early‐onset leptomeningeal amyloidosis. J Neurol. 2019;266(1):232‐241. 30470998 10.1007/s00415-018-9125-z

[26] Guo T, Tan ZP, Chen HM, et al. An effective combination of whole‐exome sequencing and runs of homozygosity for the diagnosis of primary ciliary dyskinesia in consanguineous families. Sci Rep. 2017;7(1):7905. 28801648 10.1038/s41598-017-08510-z PMC5554225

[27] Lim KH, Fairbrother WG. Spliceman‐‐a computational web server that predicts sequence variations in pre‐mRNA splicing. Bioinformatics. 2012;28(7):1031‐1032. 22328782 10.1093/bioinformatics/bts074 PMC3315715

[28] Xiang Q, Cao Y, Xu H, et al. Identification of novel pathogenic ABCA4 variants in a Han Chinese family with Stargardt disease. Biosci Rep. 2019;39(1):BSR20180872. 30563929 10.1042/BSR20180872 PMC6331664

[29] Richards S, Aziz N, Bale S, et al. Standards and guidelines for the interpretation of sequence variants: a joint consensus recommendation of the American College of Medical Genetics and Genomics and the Association for Molecular Pathology. Genet Med. 2015;17(5):405‐424. 25741868 10.1038/gim.2015.30 PMC4544753

[30] Behan L, Dimitrov BD, Kuehni CE, et al. PICADAR: a diagnostic predictive tool for primary ciliary dyskinesia. Eur Respir J. 2016;47(4):1103‐1112. 26917608 10.1183/13993003.01551-2015 PMC4819882

[31] Boon M, Meyts I, Proesmans M, et al. Diagnostic accuracy of nitric oxide measurements to detect primary ciliary dyskinesia. Eur J Clin Invest. 2014;44(5):477‐485. 24597492 10.1111/eci.12254

[32] Yuan L, Guo Y, Yi J, et al. Identification of a novel GJA3 mutation in congenital nuclear cataract. Optom Vis Sci. 2015;92(3):337‐342. 25635993 10.1097/OPX.0000000000000518

[33] Zheng W, Chen H, Deng X, et al. Identification of a novel mutation in the titin gene in a Chinese family with limb‐girdle muscular dystrophy 2J. Mol Neurobiol. 2016;53(8):5097‐5102. 26392295 10.1007/s12035-015-9439-0

[34] Højgaard C, Kofoed C, Espersen R, et al. A soluble, folded protein without charged amino acid residues. Biochemistry. 2016;55(28):3949‐3956. 27307139 10.1021/acs.biochem.6b00269

[35] Damseh N, Quercia N, Rumman N, et al. Primary ciliary dyskinesia: mechanisms and management. Appl Clin Genet. 2017;10:67‐74. 29033599 10.2147/TACG.S127129 PMC5614735

[36] Paff T, Daniels JMA, Pals G, et al. Primary ciliary dyskinesia: from diagnosis to molecular mechanisms. J Pediatr Genet. 2014;3(2):115‐127. 27625868 10.3233/PGE-14088 PMC5020995

[37] Lai M, Pifferi M, Bush A, et al. Gene editing of DNAH11 restores normal cilia motility in primary ciliary dyskinesia. J Med Genet. 2016;53(4):242‐249. 26729821 10.1136/jmedgenet-2015-103539

[38] Kim RH, Hall DA, Cutz E, et al. The role of molecular genetic analysis in the diagnosis of primary ciliary dyskinesia. Ann Am Thorac Soc. 2014;11(3):351‐359. 24498942 10.1513/AnnalsATS.201306-194OC PMC4028737

[39] Boon M, Smits A, Cuppens H, et al. Primary ciliary dyskinesia: critical evaluation of clinical symptoms and diagnosis in patients with normal and abnormal ultrastructure. Orphanet J Rare Dis. 2014;9:11. 24450482 10.1186/1750-1172-9-11 PMC4016480

[40] Knowles MR, Leigh MW, Carson JL, et al. Mutations of DNAH11 in patients with primary ciliary dyskinesia with normal ciliary ultrastructure. Thorax. 2012;67(5):433‐441. 22184204 10.1136/thoraxjnl-2011-200301 PMC3739700

[41] Shirlow R, Fitzgerald DA. PRO: primary Ciliary dyskinesia: genes are all you need! Paediatr Resspir Rev. 2020;37:32‐33. 10.1016/j.prrv.2020.04.005 32653464

[42] Dougherty GW, Loges NT, Klinkenbusch JA, et al. DNAH11 localization in the proximal region of respiratory cilia defines distinct outer dynein arm complexes. Am J Respir Cell Mol Biol. 2016;55(2):213‐224. 26909801 10.1165/rcmb.2015-0353OC PMC4979367

[43] Wu C, Jin X, Tsueng G, et al. BioGPS: building your own mash‐up of gene annotations and expression profiles. Nucleic Acids Res. 2016;44(D1):D313‐D316. 26578587 10.1093/nar/gkv1104 PMC4702805

[44] Álvarez‐Satta M, Castro‐Sánchez S, Pousada G, et al. Functional analysis by minigene assay of putative splicing variants found in Bardet‐Biedl syndrome patients. J Cell Mol Med. 2017;21(10):2268‐2275. 28502102 10.1111/jcmm.13147 PMC5618670

[45] Millat G, Lafont E, Nony S, et al. Functional characterization of putative novel splicing mutations in the cardiomyopathy‐causing genes. DNA Cell Biol. 2015;34(7):489‐496. 25849606 10.1089/dna.2015.2842

[46] Shoemark A, Burgoyne T, Kwan R, et al. Primary ciliary dyskinesia with normal ultrastructure: three‐dimensional tomography detects absence of DNAH11. Eur Respir J. 2018;51(2):1701809. 29467202 10.1183/13993003.01809-2017

[47] Pifferi M, Michelucci A, Conidi ME, et al. New DNAH11 mutations in primary ciliary dyskinesia with normal axonemal ultrastructure. Eur Respir J. 2010;35(6):1413‐1416. 20513915 10.1183/09031936.00186209

[48] Bartoloni L, Blouin JL, Pan Y, et al. Mutations in the DNAH11 (axonemal heavy chain dynein type 11) gene cause one form of situs inversus totalis and most likely primary ciliary dyskinesia. Proc Natl Acad Sci U S A. 2002;99(16):10282‐10286. 12142464 10.1073/pnas.152337699 PMC124905

[49] Zhu D, Zhang H, Wang R, et al. Association of DNAH11 gene polymorphisms with asthenozoospermia in Northeast Chinese patients. Biosci Rep. 2019;39(6):BSR20181450. 31160482 10.1042/BSR20181450 PMC6617048

[50] Zuccarello D, Ferlin A, Cazzadore C, et al. Mutations in dynein genes in patients affected by isolated non‐syndromic asthenozoospermia. Hum Repord. 2008;23(8):1957‐1962. 10.1093/humrep/den193 18492703

[51] Edamatsu M. Functional characterization of lethal P‐loop mutations in Tetrahymena outer arm dynein (Dyh3p). Biochem Biophys Res Commun. 2018;496(4):1382‐1388. 29425819 10.1016/j.bbrc.2018.02.038

[52] Silvanovich A, Li MG, Serr M, et al. The third P‐loop domain in cytoplasmic dynein heavy chain is essential for dynein motor function and ATP‐sensitive microtubule binding. Mol Biol Cell. 2003;14(4):1355‐1365. 12686593 10.1091/mbc.E02-10-0675 PMC153106

[53] Furuta A, Yagi T, Yanagisawa HA, et al. Systematic comparison of in vitro motile properties between Chlamydomonas wild‐type and mutant outer arm dyneins each lacking one of the three heavy chains. J Biol Chem. 2009;284(9):5927‐5935. 19124458 10.1074/jbc.M807830200

[54] Lucas JS, Adam EC, Goggin PM, et al. Static respiratory cilia associated with mutations in Dnahc11/DNAH11: a mouse model of PCD. Hum Mutat. 2012;33(3):495‐503. 22102620 10.1002/humu.22001

[55] Supp DM, Witte DP, Potter SS, et al. Mutation of an axonemal dynein affects left‐right asymmetry in inversus viscerum mice. Nature. 1997;389(6654):963‐966. 9353118 10.1038/40140 PMC1800588

[56] Asai DJ, Koonce MP. The dynein heavy chain: structure, mechanics and evolution. Trend Cell Biol. 2001;11(5):196‐202. 10.1016/s0962-8924(01)01970-5 11316608

[57] Whitfield M, Thomas L, Bequignon E, et al. Mutations in DNAH17, encoding a sperm‐specific axonemal outer dynein arm heavy chain, cause isolated male infertility due to asthenozoospermia. Am J Hum Genet. 2019;105(1):198‐212. 31178125 10.1016/j.ajhg.2019.04.015 PMC6612517

