# Supplementary material for: Identification of compound heterozygous DNAH11 variants in a Han‐Chinese family with primary ciliary dyskinesia
Source: J Cell Mol Med. 2021 Aug 18;25(18):9028–37. doi: 10.1111/jcmm.16866 (PMC8435457; doi:10.1111/jcmm.16866)

**Figure S1. The flowsheet of variant filtering.** BDGP/NNSplice, Berkeley Drosophila Genome Project/Splice Site Prediction by Neural Network; *DNAH11*, the dynein axonemal heavy chain 11 gene; Indels, insertions-deletions; NHLBI ESP6500, NHLBI exome sequencing project 6500; PCD, primary ciliary dyskinesia; PolyPhen-2, Polymorphism Phenotyping version 2; SIFT, Sorting Intolerant from Tolerant; SNPs, single nucleotide polymorphisms.


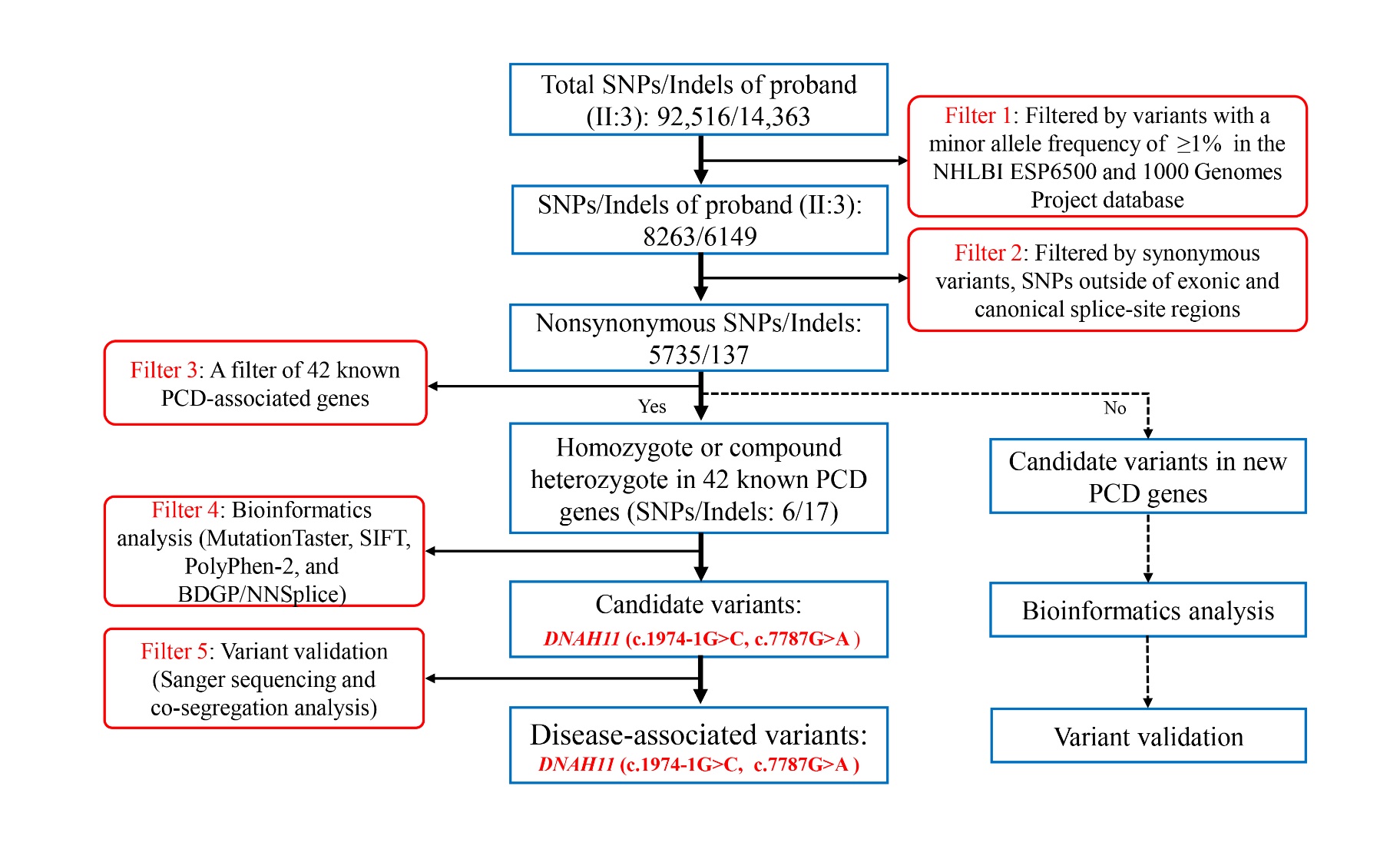

Supplement: Supplementary file 1 — Fig S1 [file JCMM-25-9028-s001.docx]
