# Supplementary material for: Identification of compound heterozygous DNAH11 variants in a Han‐Chinese family with primary ciliary dyskinesia
Source: J Cell Mol Med. 2021 Aug 18;25(18):9028–37. doi: 10.1111/jcmm.16866 (PMC8435457; doi:10.1111/jcmm.16866)
Supplement: Supplementary file 2 — Table S1 [file JCMM-25-9028-s003.docx]

**Table S1.** **The reported PCD-associated genes.**

| Clinical phenotype | Ciliary ultrastructural phenotype | PCD gene | Gene full name | OMIM number | Reference |
| --- | --- | --- | --- | --- | --- |
| PCD with laterality defects | Normal axoneme  ultrastructure | *DNAH11* | The dynein axonemal heavy chain 11 gene | 603339 | (1) |
|  |  | *OFD1* | The OFD1 centriole and centriolar satellite protein gene | 300170 | (2) |
|  |  | *GAS2L2* | The growth arrest specific 2 like 2 gene | 611398 | (3) |
|  | ODA defects | *DNAH5* | The dynein axonemal heavy chain 5 gene | 603335 | (4,5) |
|  |  | *DNAH9* | The dynein axonemal heavy chain 9 gene | 603330 | (6,7) |
|  |  | *DNAI1* | The dynein axonemal intermediate chain 1 gene | 604366 | (8) |
|  |  | *DNAI2* | The dynein axonemal intermediate chain 2 gene | 605483 | (9) |
|  |  | *DNAL1* | The dynein axonemal light chain 1 gene | 610062 | (10) |
|  |  | *LRRC56* | The leucine rich repeat containing 56 gene | 618227 | (11) |
|  |  | *NME8* | The NME/NM23 family member 8 gene | 607421 | (12) |
|  |  | *ODAD1* | The outer dynein arm docking complex subunit 1 gene | 615038 | (13,14) |
|  |  | *ODAD2* | The outer dynein arm docking complex subunit 2 gene | 615408 | (15) |
|  |  | *ODAD3* | The outer dynein arm docking complex subunit 3 gene | 615956 | (16) |
|  |  | *ODAD4* | The outer dynein arm docking complex subunit 4 gene | 617095 | (17) |
|  | ODA and IDA defects | *CCDC103* | The coiled-coil domain containing 103 gene | 614677 | (18,19) |
|  |  | *DNAAF1* | The dynein axonemal assembly factor 1 gene | 613190 | (20,21) |
|  |  | *DNAAF2* | The dynein axonemal assembly factor 2 gene | 612517 | (22) |
|  |  | *DNAAF3* | The dynein axonemal assembly factor 3 gene | 614566 | (23) |
|  |  | *DNAAF4* | The dynein axonemal assembly factor 4 gene | 608706 | (24) |
|  |  | *DNAAF5* | The dynein axonemal assembly factor 5 gene | 614864 | (25) |
|  |  | *DNAAF6* | The dynein axonemal assembly factor 6 gene | 300933 | (26,27) |
|  |  | *DNAAF11* | The dynein axonemal assembly factor 11 gene | 614930 | (28) |
|  |  | *ZMYND10* | The zinc finger MYND-type containing 10 gene | 607070 | (29,30) |
|  |  | *SPAG1* | The sperm associated antigen 1 gene | 603395 | (31) |
|  |  | *CFAP298* | The cilia and flagella associated protein 298 gene | 615494 | (32) |
|  |  | *CFAP300* | The cilia and flagella associated protein 300 gene | 618058 | (33) |
|  | IDA defects and axonemal disorganization | *CCDC39* | The coiled-coil domain containing 39 gene | 613798 | (34) |
|  |  | *CCDC40* | The coiled-coil domain containing 40 gene | 613799 | (35) |
|  | Variable ultrastructural abnormalities | *FOXJ1* | The forkhead box J1 gene | 602291 | (36) |
| PCD without laterality defects | Central-complex and radial spoke defects | *RSPH1* | The radial spoke head component 1 gene | 609314 | (37) |
|  |  | *RSPH3* | The radial spoke head 3 gene | 615876 | (38) |
|  |  | *RSPH4A* | The radial spoke head component 4A gene | 612647 | (39) |
|  |  | *RSPH9* | The radial spoke head component 9 gene | 612648 | (39) |
|  |  | *DNAJB13* | The DnaJ heat shock protein family (Hsp40) member B13 gene | 610263 | (40) |
|  | Central pair associated protein defects | *HYDIN* | The HYDIN axonemal central pair apparatus protein gene | 610812 | (41) |
|  |  | *STK36* | The serine/threonine kinase 36 gene | 607652 | (42) |
|  | Isolated nexin link defects | *DRC1* | The dynein regulatory complex subunit 1 gene | 615288 | (43) |
|  |  | *CCDC65* | The coiled-coil domain containing 65 gene | 611088 | (32) |
|  |  | *GAS8* | The growth arrest specific 8 gene | 605178 | (44,45) |
|  | Basal body abnormalities | *MCIDAS* | The multiciliate differentiation and DNA synthesis associated cell cycle protein gene | 614086 | (46) |
|  |  | *CCNO* | The cyclin O gene | 607752 | (47) |
|  | Normal axoneme  ultrastructure | *NEK10* | The NIMA related kinase 10 gene | 618726 | (48) |

PCD, primary ciliary dyskinesia; ODA, outer dynein arm; IDA, inner dynein arm; OMIM, Online Mendelian Inheritance in Man.

**REFERENCES**

1. Knowles MR, Leigh MW, Carson JL, et al. Mutations of DNAH11 in patients with primary ciliary dyskinesia with normal ciliary ultrastructure. *Thorax.* 2012;67(5):433-441.

2. Bukowy-Bieryllo Z, Rabiasz A, Dabrowski M, et al. Truncating mutations in exons 20 and 21 of OFD1 can cause primary ciliary dyskinesia without associated syndromic symptoms. *J Med Genet.* 2019;56(11):769-777.

3. Bustamante-Marin XM, Yin WN, Sears PR, et al. Lack of GAS2L2 causes PCD by impairing cilia orientation and mucociliary clearance. *Am J Hum Genet.* 2019;104(2):229-245.

4. Hornef N, Olbrich H, Horvath J, et al. DNAH5 mutations are a common cause of primary ciliary dyskinesia with outer dynein arm defects. *Am J Respir Crit Care Med.* 2006;174(2):120-126.

5. Olbrich H, Häffner K, Kispert A, et al. Mutations in DNAH5 cause primary ciliary dyskinesia and randomization of left-right asymmetry. *Nat Genet.* 2002;30(2):143-144.

6. Fassad MR, Shoemark A, Legendre M, et al. Mutations in outer dynein arm heavy chain DNAH9 cause motile cilia defects and situs inversus. *Am J Hum Genet.* 2018;103(6):984-994.

7. Loges NT, Antony D, Maver A, et al. Recessive DNAH9 loss-of-function mutations cause laterality defects and subtle respiratory ciliary-beating defects. *Am J Hum Genet.* 2018;103(6):995-1008.

8. Pennarun G, Escudier E, Chapelin C, et al. Loss-of-function mutations in a human gene related to Chlamydomonas reinhardtii dynein IC78 result in primary ciliary dyskinesia. *Am J Hum Genet.* 1999;65(6):1508-1519.

9. Loges NT, Olbrich H, Fenske L, et al. DNAI2 mutations cause primary ciliary dyskinesia with defects in the outer dynein arm. *Am J Hum Genet.* 2008;83(5):547-558.

10. Mazor M, Alkrinawi S, Chalifa-Caspi V, et al. Primary ciliary dyskinesia caused by homozygous mutation in DNAL1, encoding dynein light chain 1. *Am J Hum Genet.* 2011;88(5):599-607.

11. Bonnefoy S, Watson CM, Kernohan KD, et al. Biallelic mutations in LRRC56, encoding a protein associated with intraflagellar transport, cause mucociliary clearance and laterality defects. *Am J Hum Genet.* 2018;103(5):727-739.

12. Duriez B, Duquesnoy P, Escudier E, et al. A common variant in combination with a nonsense mutation in a member of the thioredoxin family causes primary ciliary dyskinesia. *Proc Natl Acad Sci U S A.* 2007;104(9):3336-3341.

13. Knowles MR, Leigh MW, Ostrowski LE, et al. Exome sequencing identifies mutations in CCDC114 as a cause of primary ciliary dyskinesia. *Am J Hum Genet.* 2013;92(1):99-106.

14. Onoufriadis A, Paff T, Antony D, et al. Splice-site mutations in the axonemal outer dynein arm docking complex gene CCDC114 cause primary ciliary dyskinesia. *Am J Hum Genet.* 2013;92(1):88-98.

15. Hjeij R, Lindstrand A, Francis R, et al. ARMC4 mutations cause primary ciliary dyskinesia with randomization of left/right body asymmetry. *Am J Hum Genet.* 2013;93(2):357-367.

16. Hjeij R, Onoufriadis A, Watson CM, et al. CCDC151 mutations cause primary ciliary dyskinesia by disruption of the outer dynein arm docking complex formation. *Am J Hum Genet.* 2014;95(3):257-274.

17. Wallmeier J, Shiratori H, Dougherty GW, et al. TTC25 deficiency results in defects of the outer dynein arm docking machinery and primary ciliary dyskinesia with left-right body asymmetry randomization. *Am J Hum Genet.* 2016;99(2):460-469.

18. Panizzi JR, Becker-Heck A, Castleman VH, et al. CCDC103 mutations cause primary ciliary dyskinesia by disrupting assembly of ciliary dynein arms. *Nat Genet.* 2012;44(6):714-719.

19. Shoemark A, Moya E, Hirst RA, et al. High prevalence of CCDC103 p.His154Pro mutation causing primary ciliary dyskinesia disrupts protein oligomerisation and is associated with normal diagnostic investigations. *Thorax.* 2018;73(2):157-166.

20. Loges NT, Olbrich H, Becker-Heck A, et al. Deletions and point mutations of LRRC50 cause primary ciliary dyskinesia due to dynein arm defects. *Am J Hum Genet.* 2009;85(6):883-889.

21. Duquesnoy P, Escudier E, Vincensini L, et al. Loss-of-function mutations in the human ortholog of Chlamydomonas reinhardtii ODA7 disrupt dynein arm assembly and cause primary ciliary dyskinesia. *Am J Hum Genet.* 2009;85(6):890-896.

22. Omran H, Kobayashi D, Olbrich H, et al. Ktu/PF13 is required for cytoplasmic pre-assembly of axonemal dyneins. *Nature.* 2008;456(7222):611-616.

23. Mitchison HM, Schmidts M, Loges NT, et al. Mutations in axonemal dynein assembly factor DNAAF3 cause primary ciliary dyskinesia. *Nat Genet.* 2012;44(4):381-389.

24. Tarkar A, Loges NT, Slagle CE, et al. DYX1C1 is required for axonemal dynein assembly and ciliary motility. *Nat Genet.* 2013;45(9):995-1003.

25. Horani A, Druley TE, Zariwala MA, et al. Whole-exome capture and sequencing identifies HEATR2 mutation as a cause of primary ciliary dyskinesia. *Am J Hum Genet.* 2012;91(4):685-693.

26. Paff T, Loges NT, Aprea I, et al. Mutations in PIH1D3 cause X-linked primary ciliary dyskinesia with outer and inner dynein arm defects. *Am J Hum Genet.* 2017;100(1):160-168.

27. Olcese C, Patel MP, Shoemark A, et al. X-linked primary ciliary dyskinesia due to mutations in the cytoplasmic axonemal dynein assembly factor PIH1D3. *Nat Commun.* 2017;8:14279.

28. Kott E, Duquesnoy P, Copin B, et al. Loss-of-function mutations in LRRC6, a gene essential for proper axonemal assembly of inner and outer dynein arms, cause primary ciliary dyskinesia. *Am J Hum Genet.* 2012;91(5):958-964.

29. Zariwala MA, Gee HY, Kurkowiak M, et al. ZMYND10 is mutated in primary ciliary dyskinesia and interacts with LRRC6. *Am J Hum Genet.* 2013;93(2):336-345.

30. Moore DJ, Onoufriadis A, Shoemark A, et al. Mutations in ZMYND10, a gene essential for proper axonemal assembly of inner and outer dynein arms in humans and flies, cause primary ciliary dyskinesia. *Am J Hum Genet.* 2013;93(2):346-356.

31. Knowles MR, Ostrowski LE, Loges NT, et al. Mutations in SPAG1 cause primary ciliary dyskinesia associated with defective outer and inner dynein arms. *Am J Hum Genet.* 2013;93(4):711-720.

32. Austin-Tse C, Halbritter J, Zariwala MA, et al. Zebrafish ciliopathy screen plus human mutational analysis identifies c21orf59 and CCDC65 defects as causing primary ciliary dyskinesia. *Am J Hum Genet.* 2013;93(4):672-686.

33. Höben IM, Hjeij R, Olbrich H, et al. Mutations in c11orf70 cause primary ciliary dyskinesia with randomization of left/right body asymmetry due to defects of outer and inner dynein arms. *Am J Hum Genet.* 2018;102(5):973-984.

34. Merveille A, Davis EE, Becker-Heck A, et al. CCDC39 is required for assembly of inner dynein arms and the dynein regulatory complex and for normal ciliary motility in humans and dogs. *Nat Genet.* 2011;43(1):72-78.

35. Becker-Heck A, Zohn IE, Okabe N, et al. The coiled-coil domain containing protein CCDC40 is essential for motile cilia function and left-right axis formation. *Nat Genet.* 2011;43(1):79-84.

36. Wallmeier J, Frank D, Shoemark A, et al. De novo mutations in FOXJ1 result in a motile ciliopathy with hydrocephalus and randomization of left/right body asymmetry. *Am J Hum Genet*. 2019;105(5):1030-1039.

37. Kott E, Legendre M, Copin B, et al. Loss-of-function mutations in RSPH1 cause primary ciliary dyskinesia with central-complex and radial-spoke defects. *Am J Hum Genet.* 2013;93(3):561-570.

38. Jeanson L, Copin B, Papon J, et al. RSPH3 mutations cause primary ciliary dyskinesia with central-complex defects and a near absence of radial spokes. *Am J Hum Genet*. 2015;97(1):153-162.

39. Castleman VH, Romio L, Chodhari R, et al. Mutations in radial spoke head protein genes RSPH9 and RSPH4A cause primary ciliary dyskinesia with central-microtubular-pair abnormalities. *Am J Hum Genet*. 2009;84(2):197-209.

40. El Khouri E, Thomas L, Jeanson L, et al. Mutations in DNAJB13, encoding an HSP40 family member, cause primary ciliary dyskinesia and male infertility. *Am J Hum Genet*. 2016;99(2):489-500.

41. Olbrich H, Schmidts M, Werner C, et al. Recessive HYDIN mutations cause primary ciliary dyskinesia without randomization of left-right body asymmetry. *Am J Hum Genet*. 2012;91(4):672-684.

42. Edelbusch C, Cindrić S, Dougherty GW, et al. Mutation of serine/threonine protein kinase 36 (STK36) causes primary ciliary dyskinesia with a central pair defect. *Hum Mutat.* 2017;38(8):964-969.

43. Wirschell M, Olbrich H, Werner C, et al. The nexin-dynein regulatory complex subunit DRC1 is essential for motile cilia function in algae and humans. *Nat Genet.* 2013;45(3):262-268.

44. Olbrich H, Cremers C, Loges NT, et al. Loss-of-function GAS8 mutations cause primary ciliary dyskinesia and disrupt the nexin-dynein regulatory complex. *Am J Hum Genet*. 2015;97(4):546-554.

45. Jeanson L, Thomas L, Copin B, et al. Mutations in GAS8, a gene encoding a nexin-dynein regulatory complex subunit, cause primary ciliary dyskinesia with axonemal disorganization. *Hum Mutat.* 2016;37(8):776-785.

46. Boon M, Wallmeier J, Ma L, et al. MCIDAS mutations result in a mucociliary clearance disorder with reduced generation of multiple motile cilia. *Nat Commun.* 2014;5:4418.

47. Wallmeier J, Al-Mutairi DA, Chen CT, et al. Mutations in CCNO result in congenital mucociliary clearance disorder with reduced generation of multiple motile cilia. *Nat Genet.* 2014;46(6):646-651.

48. Chivukula RR, Montoro DT, Leung HM, et al. A human ciliopathy reveals essential functions for NEK10 in airway mucociliary clearance. *Nat Med.* 2020;26(2):244-251.
