# Supplementary material for: Identification of compound heterozygous DNAH11 variants in a Han‐Chinese family with primary ciliary dyskinesia
Source: J Cell Mol Med. 2021 Aug 18;25(18):9028–37. doi: 10.1111/jcmm.16866 (PMC8435457; doi:10.1111/jcmm.16866)
Supplement: Supplementary file 3 — Table S2 [file JCMM-25-9028-s002.doc]

**Table S2. Genotype-phenotype correlation analysis of PCD individuals with *DNAH11* variants.**

| Patients | Sex | Nucleotide change^#^ | Amino acid change^#^ | Zygosity | NRD | RRI | Bxsis | Rhinitis | Sinusitis | Otitis media | Subfertility | Situs | TEM | References |
| --- | --- | --- | --- | --- | --- | --- | --- | --- | --- | --- | --- | --- | --- | --- |
| - | F | c.73G>A  c.5702A>C | p.(Ala25Thr)  p.(Glu1901Ala) | CH | NA | + | + | + | + | NA | NA | SI | NA | (1) |
| HCMJ20 | NA | c.122_147del  c.11853del | p.(Asn41Serfs*56)  p.(Phe3952Leufs*13) | CH | NA | NA | NA | NA | NA | NA | NA | SI | NA | (2) |
| OP41-II:1 | M | c.350A>T  c.7148T>C | p.(Glu117Val)  p.(Leu2383Pro) | CH | + | NA | NA | NA | + | + | NA | SI | Normal | (3) |
| P44 | M | c.846G>C  c.2406G>A | p.(Met282Ile)  p.(Trp802*) | CH | - | - | - | NA | + | NA | NA | SA | Normal | (4) |
| A  B | F  M | c.883-1G>A  c.4130G>A | NA  p.(Trp1377*) | CH | - | + | NA | +  - | +  - | +  - | NA | SI | Normal | (5) |
| 11182 | F | c.1848G>A^§^ | p.(Gln616Gln) | Het | - | + | + | + | + | + | NA | SS | Normal | (6) |
| II:3 | M | c.1974-1G>C  c.7787G>A | NA  p.(Arg2596Gln) | CH | - | + | + | + | + | + | - | SI | NA | Current study |
| PCD761 | F | c.2275-1G>C  c.13213del | p.Tys759_Glu889del  p.(Arg4405Alafs*2) | CH | + | NA | + | NA | + | + | NA | SI | Normal | (3) |
| 1  2 | F  M | c.2341G>A  c.7645+5G>A | p.(Glu781Lys)  NA | CH | - | + | - | + | + | +  - | NA | SI  SS | NA | (7) |
| 3  4  5 | F | c.2341G>A | p.(Glu781Lys) | Hom | - | + | - | +  -  - | - | + | NA | SS  SI  SS | NA | (7) |
| P28 | M | c.2485C>T  c.5608C>T | p.(Arg829Cys)  p.(Pro1870Ser) | CH | - | + | NA | NA | + | + | NA | SS | NA | (4) |
| #394 | NA | c.2491C>T  c.3871G>A | p.(Gln831*)  p.(Ala1291Thr) | CH | NA | NA | NA | NA | NA | NA | NA | SI | Normal | (8) |
| PCD1174 | F | c.2569C>T | p.(Arg857*) | Het | + | NA | + | NA | + | + | NA | SS | NA | (3) |
| 41 | F | c.2569C>T  c.4456_4458delinsAT | p.(Arg857*)  p.(Leu1486Ilefs*11) | CH | + | NA | - | NA | + | + | NA | SI | Normal | (9) |
| HCMJ92 | NA | NA | p.(Lys896fs) | Hom | NA | NA | NA | NA | NA | NA | NA | SS | NA | (2) |
| HCMJ2 | NA | NA  c.4333C>T | p.(Trp904*)  p.(Arg1445*) | CH | NA | NA | NA | NA | NA | NA | NA | SS | NA | (2) |
| 51 | F | c.2750A>T | p.(Glu917Val) | Hom | NA | NA | NA | NA | NA | NA | NA | SS | Inconclusive | (10) |
| 11174 | M | c.2753G>T  c.12796_12801delinsATA | p.(Gly918Val)  p.(Phe4266_Asn4267delinsIle) | CH | - | + | - | + | + | + | NA | SS | Normal | (6) |
| #4 | F | c.2832dup  c.13240dup | p.(Gln945Serfs*10)  p.(Thr4414Asnfs*34) | CH | NA | + | NA | + | NA | NA | NA | SS | Normal | (11) |
| HCMJ95 | NA | NA  c.9436G>T | p.(Pro959fs)  p.(Glu3146*) | CH | NA | NA | NA | NA | NA | NA | NA | SS | NA | (2) |
| HCMJ105 | NA | c.2965C>T  c.12358C>T | p.(Arg989*)  p.(Leu4120Phe) | CH | NA | NA | NA | NA | NA | NA | NA | SI | NA | (2) |
| P25 | M | c.3020T>G  c.3470T>G | p.(Leu1007*)  p.(Leu1157Arg) | CH | - | + | - | NA | + | + | NA | SI | Normal | (4) |
| #6 | F | c.3220G>T  c.13069C>T | p.(Glu1074*)  p.(Arg4357*) | CH | NA | + | NA | - | NA | NA | NA | SI | Normal | (11) |
| HCMJ133 | NA | c.3356G>A  NA  NA | p.(Ser1119Asn)  p.(Trp1222Arg)  p.(Phe4266_Asn4267delinsIle) | CH | NA | NA | NA | NA | NA | NA | NA | SI | NA | (2) |
| P45 | F | c.3470T>G  c.6727C>G | p.(Leu1157Arg)  p.(Arg2243Gly) | CH | - | + | + | NA | + | + | NA | SI | Normal | (4) |
| KT2 | M | c.3470T>G | p.(Leu1157Arg) | Het | NA | + | + | - | - | NA | NA | SI | NA | (12) |
| #7 | M | c.3544C>T  c.8798-5G>A | p.(Arg1182*)  NA | CH | NA | + | NA | + | NA | NA | NA | SS | Normal | (11) |
| #1 | M | c.3727G>T  c.13531_13532insTTCAGGCTGAAGA | p.(Glu1243*)  p.(Ala4511Valfs*13) | CH | NA | + | NA | + | NA | NA | NA | SS | Normal | (11) |
| PCD1077 | F | c.3901G>T  c.11804C>T | p.(Glu1301*)  p.(Pro3935Leu) | CH | + | NA | NA | NA | + | + | NA | SI | Normal | (3) |
| OP406-II:2 | F | c.4254+5G>T  c.4726-1G>A | p.Glu1366_Gly1418del  p.Glu1576Alafs*4 | CH | + | NA | NA | NA | + | NA | NA | SS | Normal | (3) |
| 52  PCD1022 | F  M | c.4333C>T | p.(Arg1445*) | Hom | -  + | NA | +  - | NA | + | + | NA | SI  SS | Inadequate  Normal | (9)  (3) |
| HCMJ101 | NA | c.4333C>T  c.6203G>A  NA | p.(Arg1445*)  p.(Arg2068His)  p.(Trp3238Arg) | CH | NA | NA | NA | NA | NA | NA | NA | SS | NA | (2) |
| #560 | NA | c.4438C>T  c.8698C>T | p.(Arg1480*)  p.(Arg2900*) | CH | NA | NA | NA | NA | NA | NA | NA | SI | Normal | (8) |
| PCD623 | F | c.4438C>T | p.(Arg1480*) | Hom | + | NA | + | NA | + | + | NA | SS | Normal | (3) |
| P32 | M | c.4457T>A  c.10006G>T | p.(Leu1486Gln)  p.(Ala3336Ser) | CH | + | + | - | NA | + | + | NA | SS | Inconclusive | (4) |
| 9003 | F | c.4505A>C  c.9376G>A | p.(Gln1502Pro)  p.(Glu3126Lys) | CH | + | + | + | NA | + | + | NA | SA | Normal | (13) |
| PCD108 | M | c.4516_4517del  c.7266+1G>A | p.(Leu1506Serfs*11)  p.Thr2379_Gln2422del | CH | + | NA | - | NA | + | + | NA | SI | Normal | (3) |
| 3 | F | c.4610_4613del | p.(Met1537Lysfs*24) | Hom | NA | NA | + | + | + | NA | NA | NA | NA | (14) |
| OP-83 II2  OP-83 II3 | M  F | c.4775G>T  c.8589C>G | p.(Cys1592Phe)  p.(Ser2863Arg) | CH | -  NA | + | +  NA | NA | + | + | NA | SS  NA | NA | (15) |
| 11228 | M | c.4922C>G  c.9304G>A | p.(Ser1641*)  p.(Gly3102Ser) | CH | - | + | - | + | - | - | NA | SI | Normal | (6) |
| HCMJ134 | NA | c.5500C>G | p.(Arg1834Gly) | Het | NA | NA | NA | NA | NA | NA | NA | SI | NA | (2) |
| VDE | NA | c.5500C>G  c.8698C>T | p.(Arg1834Gly)  p.(Arg2900*) | CH | NA | NA | NA | NA | NA | NA | NA | SS | NA | (2) |
| #3 | F | c.5506C>T  c.5636T>A | p.(Arg1836*)  p.(Leu1879*) | CH | NA | + | NA | - | NA | NA | NA | SI | Normal | (11) |
| PCD545 | M | c.5643A>T | p.(Gln1881His) | Het | - | NA | + | NA | + | - | NA | SS | Normal | (3) |
| PCD565 | M | c.5778+1G>A  c.13061T>A | p.Val1821Thrfs*7  p.(Leu4354His) | CH | + | NA | + | NA | + | + | NA | SI | Normal | (3) |
| PCD812 | M | c.5815G>A  c.13373C>T | p.(Gly1939Arg)  p.(Pro4458Leu) | CH | + | NA | - | NA | + | + | NA | SI | NA | (3) |
| HCMJ110 | NA | c.6203G>A  c.7922T>C  NA | p.(Arg2068His)  p.(Phe2641Ser)  p.(Trp3238Arg) | CH | NA | NA | NA | NA | NA | NA | NA | SI | NA | (2) |
| HCMJ7 | NA | c.6203G>A  NA  c.13472_13541dup | p.(Arg2068His)  p.(Trp3238Arg)  p.(Glu4515Leufs*28) | CH | NA | NA | NA | NA | NA | NA | NA | SS | NA | (2) |
| PCD157 | F | c.6244C>T  c.11929G>T | p.(Arg2082*)  p.(Glu3977*) | CH | + | NA | + | NA | + | + | NA | SI | Normal | (3) |
| 4 | F | c.6273+1G>T | NA | Hom | NA | NA | + | + | + | NA | NA | NA | NA | (14) |
| #616 | NA | c.6506C>A | p.(Ser2169*) | Het | + | + | NA | + | NA | + | NA | SS | Normal | (16) |
| #5 | F | c.6506C>T | p.(Ser2169Leu) | Hom | NA | + | NA | + | NA | NA | NA | SS | Normal | (11) |
| A  B | M | c.6727C>T  c.10789C>T | p.(Arg2243*)  p.(Gln3597*) | CH | NA | + | + | + | + | + | NA | SI  SS | Normal | (17) |
| 7 | F | c.6983+1G>A | NA | Hom | NA | NA | + | + | + | NA | NA | NA | NA | (14) |
| P17  P30 | M | c.7292G>T  c.7364A>C  c.9017C>T  c.13373C>T | p.(Ser2431Ile)  p.(Asp2455Ala)  p.(Thr3006Met)  p.(Pro4458Leu) | CH | + | + | - | NA | +  NA | +  NA | NA | SI  SS | Normal | (4) |
| 32 | M | c.7544G>A | p.(Gly2515Glu) | Hom | NA | NA | + | + | + | NA | NA | NA | NA | (14) |
| CI | NA | c.7552G>A  c.7812-2A>T | p.(Val2518Ile)  NA | CH | NA | NA | NA | NA | NA | NA | NA | SS | NA | (2) |
| #2 | F | c.7642C>T  c.4395_4398del | p.(Gln2548*)  p.(Ser1465Argfs*6) | CH | NA | + | NA | + | NA | NA | NA | SS | Normal | (11) |
| #730 | NA | c.7772C>T  c.8698C>T | p.(Pro2591Leu)  p.(Arg2900*) | CH | + | + | NA | + | NA | + | NA | SS | Normal | (16) |
| OP98-II:1 | M | c.7914G>C  c.13330_13333dup | p.Trp2604*  p.(Ile4445Asnfs*4) | CH | - | NA | + | NA | + | + | NA | SI | Normal | (3) |
| C | M | c.8114A>G  c.10264G>A | p.(His2705Arg)  p.(Gly3422Arg) | CH | + | + | NA | - | - | + | NA | SI | Normal | (5) |
| HCMJ143 | NA | c.8266T>C  c.8362C>G | p.(Cys2756Arg)  p.(His2788Asp) | CH | NA | NA | NA | NA | NA | NA | NA | SS | NA | (2) |
| OP-63 II2  OP-63 II8 | M  F | c.8525G>A  c.8632C>T | p.(Arg2842Gln)  p.(Gln2878*) | CH | NA | + | NA | NA | + | + | NA | SI | NA | (15) |
| C.C. | M | c.8533C>T | p.(Arg2845*) | Hom | NA | + | NA | NA | NA | NA | NA | SI | Normal | (18) |
| 40 | M | c.8698C>T | p.(Arg2900*) | Het | + | NA | + | NA | + | + | NA | SS | Normal | (9) |
| OP-1250 II1 | F | c.8698C>T | p.(Arg2900*) | Hom | NA | + | + | NA | + | + | NA | SS | Normal | (15) |
| KJ | NA | c.8698C>T  c.9436G>T | p.(Arg2900*)  p.(Glu3146*) | CH | NA | NA | NA | NA | NA | NA | NA | SI | NA | (2) |
| DWS  DB | NA | c.8698C>T  c.10568+1G>A | p.(Arg2900*)  NA | CH | NA | NA | NA | NA | NA | NA | NA | SI  SS | NA | (2) |
| HCMJ137  VR | NA | c.8990G>A  c.10739G>A | p.(Arg2997Gln)  p.(Arg3580His) | CH | NA | NA | NA | NA | NA | NA | NA | SS | NA | (2) |
| SE  SA | NA | c.9103-2A>C  c.13309C>T | NA  p.(Arg4437Cys) | CH | NA | NA | NA | NA | NA | NA | NA | SS | NA | (2) |
| 998 | M | c.9113_9116del | p.(Lys3038Thrfs*14) | Het | - | NA | + | NA | + | + | NA | SS | Normal | (19) |
| 9026 | F | c.9182A>G | p.(Gln3061Arg) | Het | - | - | - | NA | - | - | NA | SA | Normal | (13) |
| P23 | F | c.9539T>A  c.9706C>T | p.(Leu3180*)  p.(Arg3236*) | CH | NA | NA | NA | NA | + | + | NA | SI | NA | (4) |
| PCD974 | F | c.9764T>C | p.(Leu3255Ser) | Het | - | NA | + | NA | + | + | NA | SS | Normal | (3) |
| OP-1324 II1  OP-1324 II2 | F  M | c.10036C>T | p.(Arg3346*) | Hom | + | +  - | NA | NA | + | + | NA | SI | Normal | (15) |
| 21 | M | c.10285C>A  Ex7-14 32.29 Kb dup | p.(Arg3429Ser)  NA | CH | NA | NA | NA | NA | NA | NA | NA | SI | Normal | (10) |
| 44 | M | c.10286G>T | p.(Arg3429Leu) | Hom | NA | NA | NA | NA | NA | NA | NA | SS | Normal | (10) |
| PCD1033 | F | c.10324C>T | p.(Gln3442*) | Het | + | NA | - | NA | + | + | NA | SA | Normal | (3) |
| 2 | M | c.10332+1G>A  c.12310C>T | NA  p.(Arg4104*) | CH | NA | + | + | NA | + | + | NA | SS | Normal | (20) |
| 15 | F | c.10877C>A  c.12460C>T | p.(Pro3626Gln)  p.(Arg4154Cys) | CH | + | NA | + | NA | + | + | NA | SS | ODA+IDA | (9) |
| OP20-II:1 | M | c.11663G>A | p.(Arg3888His) | Hom | - | NA | + | NA | - | + | NA | SI | NA | (3) |
| OP-327 II1  OP-327 II2  OP-327 II3  OP-327 II4 | M  F  M  M | c.11839+1G>A | NA | Hom | -  -  -  + | + | +  -  +  - | NA | + | +  +  -  - | NA | SS  SS  SS  SI | NA | (15) |
| BC4  HCMJ91 | NA | c.11968-1G>C | NA | Hom | NA | NA | NA | NA | NA | NA | NA | SS  SI | NA | (2) |
| PCD1126 | F | c.12064G>C  c.13500_13504dup | p.(Ala4022Pro)  p.(Thr4502Argfs*15) | CH | - | NA | + | NA | + | - | NA | SS | Normal | (3) |
| 42  43 | M  F | c.12344T>G  c.12867T>A | p.(Ile4115Ser)  p.(Cys4289*) | CH | +  - | NA | - | NA | +  - | +  - | NA | SA  SI | Normal | (9) |
| II-2  II-3  II-4  II-6  II-9  II-11 | M  F  F  M  M  M | c.12363C>G  c.13531_*36del | p.Tyr4121*  p.Ala4511_Ala4516delinsGln | CH | NA | + | -  -  +  +  NA  + | NA | + | -  +  +  -  +  - | NA | SI  SS  SS  SS  SS  SS | Normal  Normal  NA  NA  Normal  Normal | (21) |
| DiSh | NA | c.12548C>T | p.(Pro4183Leu) | Het | NA | NA | NA | NA | NA | NA | NA | SS | NA | (2) |
| OP235-II:2 | F | c.12697C>T  c.12980T>C | p.(Gln4233*)  p.(Leu4327Ser) | CH | + | NA | + | NA | + | + | NA | SI | Normal | (3) |
| PCD919 | M | c.13065_13067del  c.13075C>T | p.(Leu4356del)  p.(Arg4359*) | CH | + | NA | + | NA | + | + | NA | SA | Normal | (3) |
| OI-128 II1  OI-128 II2 | F  M | c.13175C>T | p.(Thr4392Met) | Hom | - | +  - | - | NA | -  + | - | NA | SS  SI | Normal | (15) |
| 11057 | M | c.13183C>T | p.(Arg4395*) | Hom | + | + | - | + | + | + | NA | SI | IDA | (6) |
| 38 | M | c.13345C>T | p.(Arg4449Cys) | Het | NA | NA | NA | NA | NA | NA | NA | SS | Normal | (10) |

^#^ Description of the *DNAH11* variants (reference sequence: NM_001277115.2) is recalibrated following the Human Genome Variation Society nomenclature guidelines (http://varnomen.hgvs.org/).

^§^ Predicted to affect splicing.

Reported variants only described at the protein level in the literature are underlined.

Bxsis, bronchiectasis; CH, compound heterozygote; *DNAH11*, the dynein axonemal heavy chain 11 gene; F, female; Het, heterozygote; Hom, homozygote; IDA, inner dynein arm; M, male; NA, not available; NRD, neonatal respiratory distress; ODA, outer dynein arm; PCD, primary ciliary dyskinesia; RRI, recurrent respiratory infections; SA, *situs ambiguous*; SI, *situs inversus*; SS, *situs solitus*; TEM, transmission electron microscopy; +, present; -, not present.

**REFERENCES**

1. Zhang L, Feng X, Zhang J, et al. Co-occurrence of Moyamoya syndrome and Kartagener syndrome caused by the mutation of DNAH5 and DNAH11: a case report. *BMC Neurol.* 2020;20(1):314.

2. Boon M, Smits A, Cuppens H, et al. Primary ciliary dyskinesia: critical evaluation of clinical symptoms and diagnosis in patients with normal and abnormal ultrastructure. *Orphanet J Rare Dis*. 2014;9:11.

3. Knowles MR, Leigh MW, Carson JL, et al. Mutations of DNAH11 in patients with primary ciliary dyskinesia with normal ciliary ultrastructure. *Thorax.* 2012;67(5):433-441.

4. Guo Z, Chen W, Wang L, et al. Clinical and genetic spectrum of children with primary ciliary dyskinesia in China. *J Pediatr.* 2020;225:157-165.e5.

5. Pifferi M, Michelucci A, Conidi ME, et al. New DNAH11 mutations in primary ciliary dyskinesia with normal axonemal ultrastructure. *Eur Respir J.* 2010;35(6):1413-1416.

6. Boaretto F, Snijders D, Salvoro C, et al. Diagnosis of primary ciliary dyskinesia by a targeted next-generation sequencing panel: molecular and clinical findings in Italian patients. *J Mol Diagn.* 2016;18(6):912-922.

7. Schultz R, Elenius V, Lukkarinen H, et al. Two novel mutations in the DNAH11 gene in primary ciliary dyskinesia (CILD7) with considerable variety in the clinical and beating cilia phenotype. *BMC Med Genet.* 2020;21(1):237.

8. Zariwala MA, Gee HY, Kurkowiak M, et al. ZMYND10 is mutated in primary ciliary dyskinesia and interacts with LRRC6. *Am J Hum Genet.* 2013;93(2):336-345.

9. Kim RH, Hall DA, Cutz E, et al. The role of molecular genetic analysis in the diagnosis of primary ciliary dyskinesia. *Ann Am Thorac Soc.* 2014;11(3):351-359.

10. Marshall CR, Scherer SW, Zariwala MA, et al. Whole-exome sequencing and targeted copy number analysis in primary ciliary dyskinesia. *G3 (Bethesda).* 2015;5(8):1775-1781.

11. Shoemark A, Burgoyne T, Kwan R, et al. Primary ciliary dyskinesia with normal ultrastructure: three-dimensional tomography detects absence of DNAH11. *Eur Respir J.* 2018;51(2):1701809.

12. Yue Y, Huang Q, Zhu P, et al. Identification of pathogenic mutations and investigation of the NOTCH pathway activation in Kartagener syndrome. *Front Genet.* 2019;10:749.

13. Nakhleh N, Francis R, Giese RA, et al. High prevalence of respiratory ciliary dysfunction in congenital heart disease patients with heterotaxy. *Circulation.* 2012;125(18):2232-2242.

14. Emiralioğlu N, Taşkıran EZ, Koşukcu C, et al. Genotype and phenotype evaluation of patients with primary ciliary dyskinesia: first results from Turkey. *Pediatr Pulmonol.* 2019;55(2):383-393.

15. Dougherty GW, Loges NT, Klinkenbusch JA, et al. DNAH11 localization in the proximal region of respiratory cilia defines distinct outer dynein arm complexes. *Am J Respir Cell Mol Biol.* 2016;55(2):213-224.

16. Lucas JS, Adam EC, Goggin PM, et al. Static respiratory cilia associated with mutations in Dnahc11/DNAH11: a mouse model of PCD. *Hum Mutat.* 2012;33(3):495-503.

17. Lai M, Pifferi M, Bush A, et al. Gene editing of DNAH11 restores normal cilia motility in primary ciliary dyskinesia. *J Med Genet.* 2016;53(4):242-249.

18. Bartoloni L, Blouin JL, Pan Y, et al. Mutations in the DNAH11 (axonemal heavy chain dynein type 11) gene cause one form of situs inversus totalis and most likely primary ciliary dyskinesia. *Proc Natl Acad Sci U S A.* 2002;99(16):10282-10286.

19. Berg JS, Evans JP, Leigh MW, et al. Next generation massively parallel sequencing of targeted exomes to identify genetic mutations in primary ciliary dyskinesia: implications for application to clinical testing. *Genet Med.* 2011;13(3):218-229.

20. Kurokawa A, Kondo M, Orimo M, et al. Multifaceted analysis of Japanese cases of primary ciliary dyskinesia: value of immunofluorescence for ciliary protein detection in patients with DNAH5 and DNAH11 mutations. *Respir Investig*. 2021;59(4):550-554.

21. Schwabe GC, Hoffmann K, Loges NT, et al. Primary ciliary dyskinesia associated with normal axoneme ultrastructure is caused by DNAH11 mutations. *Hum Mutat.* 2008;29(2):289-298.
